# Supporting Role for GTPase Rab27a in Hepatitis C Virus RNA Replication through a Novel miR-122-Mediated Effect

**DOI:** 10.1371/journal.ppat.1005116

**Published:** 2015-08-25

**Authors:** Tzu-Chun Chen, Chung-Han Hsieh, Peter Sarnow

**Affiliations:** 1 Department of Microbiology & Immunology, School of Medicine, Stanford University, Stanford, California, United States of America; 2 Department of Neurosurgery, School of Medicine, Stanford University, Stanford, California, United States of America; University of California, San Diego, UNITED STATES

## Abstract

The small GTPase Rab27a has been shown to control membrane trafficking and microvesicle transport pathways, in particular the secretion of exosomes. In the liver, high expression of Rab27a correlates with the development of hepatocellular carcinoma. We discovered that low abundance of Rab27a resulted in decreased hepatitis C virus (HCV) RNA and protein abundances in virus-infected cells. Curiously, both cell-associated and extracellular virus yield decreased in Rab27a depleted cells, suggesting that reduced exosome secretion did not cause the observed effect. Instead, Rab27a enhanced viral RNA replication by a mechanism that involves the liver-specific microRNA miR-122. Rab27a surrounded lipid droplets and was enriched in membrane fractions that harbor viral replication proteins, suggesting a supporting role for Rab27a in viral gene expression. Curiously, Rab27a depletion decreased the abundance of miR-122, whereas overexpression of miR-122 in Rab27a-depleted cells rescued HCV RNA abundance. Because intracellular HCV RNA abundance is enhanced by the binding of two miR-122 molecules to the extreme 5’ end of the HCV RNA genome, the diminished amounts of miR-122 in Rab27a-depleted cells could have caused destabilization of HCV RNA. However, the abundance of HCV RNA carrying mutations on both miR-122-binding sites and whose stability was supported by ectopically expressed miR-122 mimetics with compensatory mutations also decreased in Rab27a-depleted cells. This result indicates that the effect of Rab27a depletion on HCV RNA abundance does not depend on the formation of 5’ terminal HCV/miR-122 RNA complexes, but that miR-122 has a Rab27a-dependent function in the HCV lifecycle, likely the downregulation of a cellular inhibitor of HCV gene expression. These findings suggest that the absence of miR-122 results in a vulnerability not only to exoribonucleases that attack the viral genome, but also to upregulation of one more cellular factor that inhibit viral gene expression.

## Author Summary

Eukaryotic cells constantly expel a variety of small vesicles that are loaded with proteins, nucleic acids and other small compounds that were produced inside the cell. One particular kind of vesicle is called exosome. Exosomes are initially located in multivesicular compartments inside cells and are docked at the cell surface membrane by the small GTPase Rab27a. In the liver, high expression of Rab27a correlates with the development of hepatocellular carcinoma, suggesting a high trafficking capacity for exosomes. Also, it has been shown that hepatitis C virus (HCV) can spread from cell to cell via exosomes. We discovered that Rab27a abundance affects HCV virion abundance that independent from its role in exosome secretion. The presence of Rab27a in membrane-enriched replication complexes and nearby lipid droplets points to functions of Rab27a in the viral life cycle. Depletion of Rab27a resulted in a lower abundance of the liver-specific microRNA miR-122. It is known that two molecules of miR-122 form an oligomeric complex with the 5’ end of the viral RNA leading to protection of the viral RNA against cellular nucleases. However, we show that the Rab27a-mediated loss of miR-122 was independent of its role in protecting the viral RNA, very likely by the downregulation of a cellular inhibitor of HCV gene expression. These findings argue for novel, hitherto undetected roles for miR-122 in the viral life cycle.

## Introduction

Hepatitis C virus (HCV) is a hepatotropic positive-sense, single-stranded RNA virus that belongs to the *Flaviviridae* family. The HCV genome is about 9.6 kb in length and encodes a polyprotein, which is cleaved into at least ten viral proteins by host and viral proteinases [1, 2]. The open reading frame is flanked by 5’ and 3’ noncoding regions, which regulate translation and replication of the viral RNA. In addition, the 5’ terminal sequences of the HCV RNA genome form an oligomeric complex with two molecules of liver-specific miR-122 [3, 4]. This complex greatly stabilizes the viral RNA from degradation by exonucleases [5, 6].

Exposure to HCV typically leads to persistent infections that cause chronic hepatitis, liver cirrhosis, and hepatocellular carcinoma [7]. An estimated 170 million people are affected by the virus, making it a serious global health burden [8]. Recently, Gilead Sciences’ sofosbuvir/ledipasvir (Harvoni) and AbbVie's paritaprevir/ritonavir/ombitasvir plus dasabuvir (Viekira Pak) were approved as the new line of interferon-free treatment regimen. In addition, Miravirsen (Santaris Pharma, Denmark), an antisense inhibitor of miR-122, showed a decrease of HCV titers in patients chronically infected with HCV in phase II clinical trials [9], demonstrating that miR-122 is a potential therapeutic host target to combat HCV. Here, we report an additional role for miR-122 in promoting HCV infection that is independent of its well-characterized 5’ end stabilization function.

Like many RNA viruses, HCV exploits membranes and the trafficking machinery of the host for viral replication [10, 11]. For example, accumulating evidence suggests that HCV can exit infected cells via the multivesicular transport system [12–15]. While these studies employed fractionation and ultrastructural approaches, evidence for the cellular origin or the mechanism of vesicle generation remains lacking. Recently, it has been reported that Rab27a modulates exosome vesicle secretion by docking multivesicular bodies to the plasma membrane [16]. Curiously, several studies have shown that Rab27a, a small GTPase, is also involved in replication of viral genomes in cells infected with human immunodeficiency virus, herpes simplex virus, hepatitis E virus and HCV [15, 17–19]. However, the mechanism by which Rab27a modulates viral genome replication remains unclear. In this study, we found that Rab27a affects HCV RNA and virion abundance by a pathway that is independent of exosome secretions. Specifically, Rab27a located to membranes that are enriched in viral replication complexes and to lipid droplets, which are sites thought to initiate packaging of the viral RNA genome. Furthermore, intracellular abundance of Rab27a affected miR-122 abundance. Curiously, Rab27a’s modulation of miR-122 was independent of miR-122’s stabilizing role of the viral RNA. Therefore, Rab27a likely downregulates, via miR-122, a cellular inhibitor of HCV gene expression.

## Results

### Rab27a-depletion decreases extracellular exosome, HCV RNA and protein abundances

**Fig 1:**
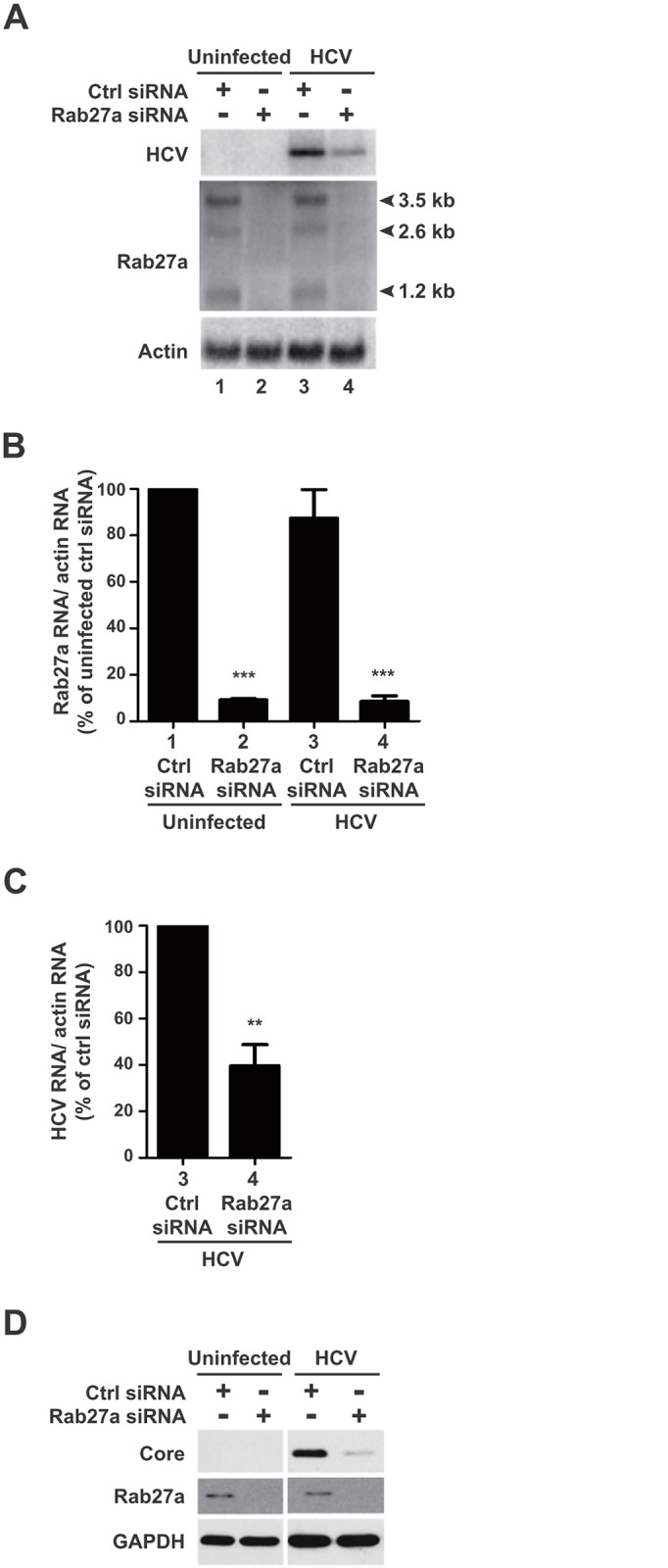
Effects of Rab27a depletion on HCV RNA and protein abundance. (A) Effects on RNA abundance. Control or Rab27a siRNA-transfected cells were infected with HCV. The abundance of Rab27a and HCV mRNA was measured by Northern blot at day 3 post-infection. Lane 1 and 2, uninfected cells; lane 3 and 4, HCV infected cells. Quantification of Rab27a (B) and HCV RNA (C) abundances are shown. RNA transcripts were normalized to actin mRNA. Data from control siRNA-treated cells was set to 100%. The data are representative of five independent replicates (**P<0.005 and ***P<0.0001, Student’s t-test). (D) Effects on protein abundance. HCV core and Rab27a proteins Abundances in Rab27a-depleted cells were examined by Western blot. The data are representative of at least three independent replicates.

To determine whether HCV RNA and protein abundances are regulated by exosomal vesicles, we first inhibited exosomal trafficking in human liver carcinoma Huh7 cells by depletion of Rab27a [16]. Northern analyses revealed that the liver Rab27a gene is transcribed into three RNA transcripts of 1.2 kb, 2.6 kb and 3.5 kb in size (Fig 1A). This result is consistent with Rab27a RNA species that are expressed in human fibrosarcoma cells [20]. All three Rab27a transcripts were decreased by 90% in both uninfected and JFH1 HCV-infected cells that were treated with siRNAs directed against the common Rab27a open reading frame (Fig 1A and 1B). As expected, Western blot analysis showed that the abundance of Rab27a protein was also decreased in Rab27a siRNA-treated cells (Fig 1D). To determine if Rab27a depletion affected extracellular exosome yield, the abundance of CD81, a marker for exosomes derived from the multivesicular body pathway, was examined in cell lysates and in extracellular, partially purified exosome preparations. S1 Fig shows that Rab27a depletion diminished the extracellular amount of CD81-containing exosomes in uninfected (S1A Fig) and in HCV-infected cells by approximately 40% (S1B Fig).

To examine the effects of Rab27a on HCV gene expression, viral RNA and protein abundances were measured in Rab27a-depleted cells. Results showed that Rab27a depletion caused a 60% decrease in HCV RNA abundance (Fig 1A, lane 4, and Fig 1C), but had no effect on actin mRNAs. Rab27a depletion also led to a decrease in HCV core protein abundance (Fig 1D). These data are consistent with a previous report on the effect of Rab27a depletion on HCV RNA abundance [15]. Similar effects of decreased viral RNA (S2A Fig) and protein (S2B Fig) protein abundances during Rab27a depletion were observed when cells were infected at a 1000-fold higher multiplicity of infection with HCV. To control for siRNA off-targeting effects, additional Rab27a siRNAs (siRNA-3 and siRNA-4), which target different regions of all Rab27a mRNA species, were tested. These siRNAs also showed decreased Rab27a and HCV RNA (S3A Fig) and protein abundances (S3B Fig). Importantly, Rab27a depletion in Huh7 cells did not have a significant effect on cell viability (S4A Fig) or caused apoptosis (S4B Fig). Therefore, depletion of Rab27a causes selective inhibition of HCV gene expression without any significant effects on cellular viability.

### Depletion of Rab27a decreases virus production

**Fig 2:**
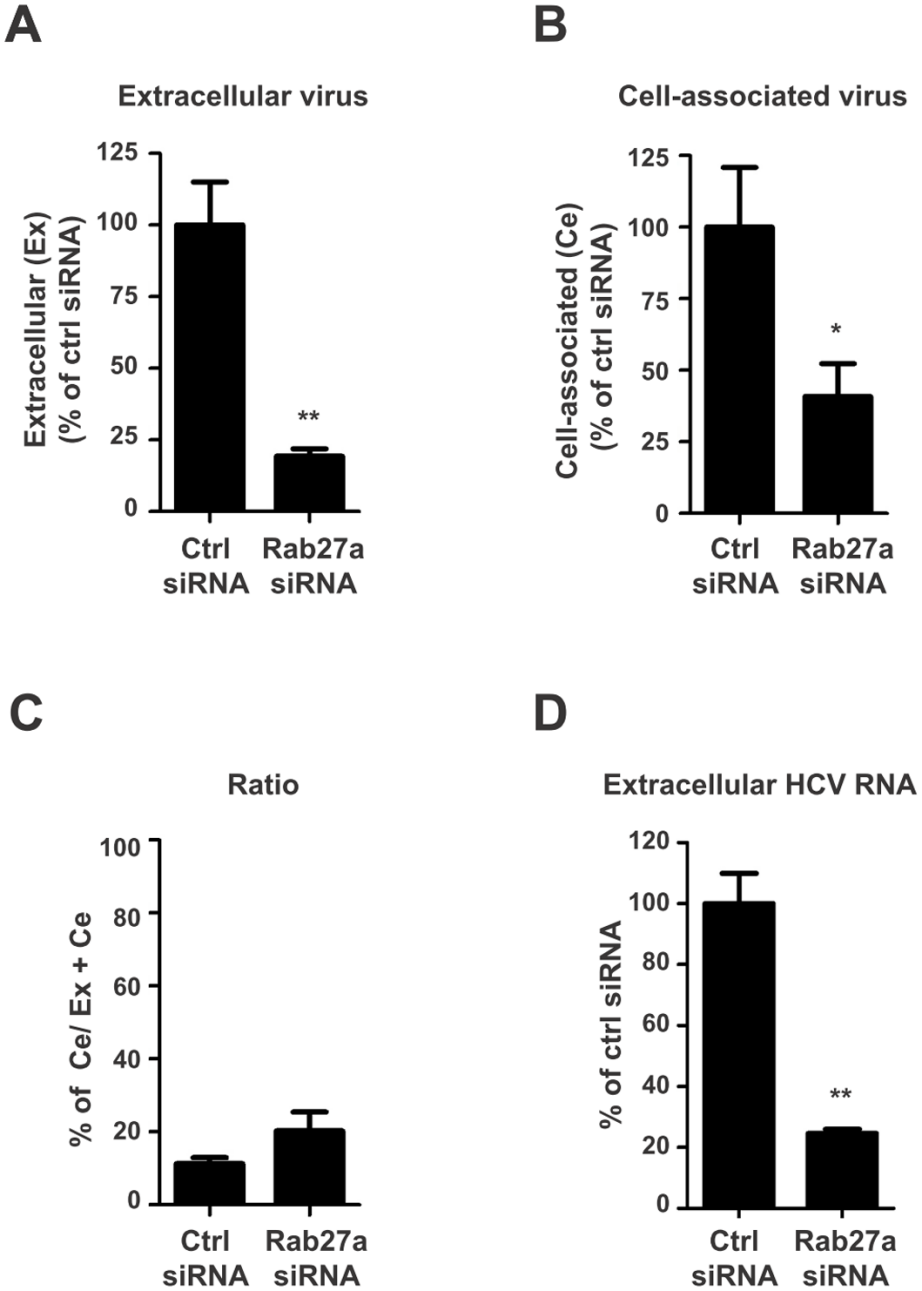
Extracellular and cell-associated virion abundances in Rab27a-depleted cells. Control or Rab27a siRNAs-treated cells were infected with HCV. Virus titers from infected cells at day 3 post-infection were determined by a fluorescent focus-forming assay. The extracellular (A) and cell-associated (B) virus titers in control siRNA-treated cells are shown with an average titer of 9.6 x 10^4^ and 2.4 x 10^4^ FFU/mL, respectively. The viral titer in cells treated with control siRNA was set to 100%. (C) Ratio of cell-associated virus to total virus particles. The cell-associated virus titer was divided by the total virus titer (extracellular plus cell-associated virus titers). (D) Extracellular HCV RNA abundance from control and Rab27a-depleted cells. The data are representative of three independent replicates (*P<0.05 and **P<0.01, Student’s t-test).

It has been reported that HCV can be transmitted from cell to cell via exosomes [12, 14, 21–23]. Rab27a plays a role in exosome secretion. Thus, we would expect an increase in cell-associated virus titer in Rab27a-siRNA treated cells compared to control-siRNA treated cells. Depletion of Rab27a decreased extracellular virus titer by about 80% (Fig 2A), but, surprisingly, cell-associated virus titer also decreased by about 60% (Fig 2B). However, the ratio of cell-associated to total infectious virus particles in Rab27a-depleted cells was similar to that of control-siRNA treated cells (Fig 2C). Consistently, extracellular HCV RNA abundance was decreased to nearly 80% in infected cells that were treated with Rab27a siRNAs, compared to Ctrl siRNAs-treated cells (4.9 x 10^6^ copies/ml) (Fig 2D). The decrease of extracellular HCV RNA abundance did not cause an accumulation of intracellular HCV (Fig 1A, lane 4, and Fig 1C). Thus, these data suggest that the diminished yield of cell-associated infectious virus particles during Rab27a depletion is not due to impaired exosome secretion, arguing that Rab27a modulates HCV gene expression by a mechanism that is different from its role in exosome secretion.

### Rab27a affects HCV gene expression

**Fig 3:**
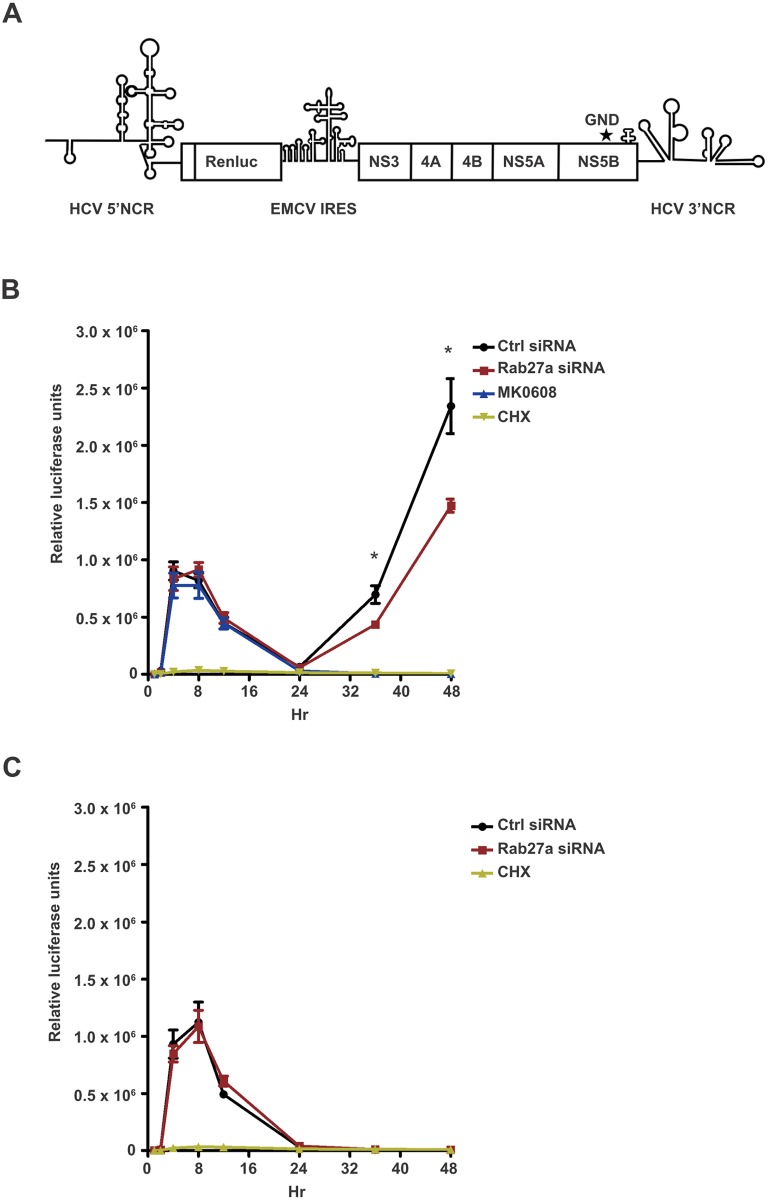
Effect of Rab27a depletion on HCV gene expression. (A) Structure of the subgenomic JFH1-Rluc replicon construct (sgJFH1-Rluc). GND mutation is marked with an asterisk. (B) Luciferase values from expressed sgJFH1-RLuc RNA in the presence and absence of Rab27a. The MK-0608 nucleoside analog was used to inhibit viral replication and cycloheximide (CHX) was used to inhibit translation. (C) Luciferase values from expressed replication-deficient sgJFH1-RLuc-GND RNA. Huh7 cells were transfected with control or Rab27a siRNAs for one day, followed by transfection of sgJFH1-RLuc (B) or sgJFH1-RLuc-GND (C) RNA. Cells were treated with cycloheximide (CHX) at 100 μg/mL or MK-0608 nucleoside analog at 50 μM. Luciferase activity was measured at 1, 2, 4, 8, 12, 24, 36 and 48 hr post-transfection. The data are representative of five independent replicates (*P<0.05, one-way Anova).

To determine whether Rab27a modulates viral RNA abundance at the RNA replication or translation step, and to bypass any effects on virion entry, we monitored the expression of subgenomic JFH1-Rluc (sgJFH1-Rluc) replicons [24, 25] (Fig 3A). These replicons are either competent for both translation and RNA replication, or contained a GND mutation in the catalytic domain of the viral RNA-dependent RNA polymerase (NS5B) that prevents genome replication (Fig 3A). Briefly, Huh7 cells were transfected with Rab27a siRNAs, and subsequently transfected with replication-competent sgJFH1-Rluc RNAs (Fig 3B) or replication-defective sgJFH1-Rluc-GND RNAs (Fig 3C). Luciferase activity was measured at different times after HCV RNA transfection. Two peaks of luciferase activity were noted in the sgJFH1-Rluc RNA-transfected cells treated with control siRNAs (Fig 3B). The first peak at 4 hours post-transfection represents the initial translation of the input RNA, which is absent in cyclocheximide-treated cells (Fig 3B and 3C). The second luciferase peak represents the translation of replicating RNAs, because it is absent in sgJFH1-Rluc-transfected cells that were treated with the NS5B inhibitor MK-0608 (Fig 3B) and in sgJFH1-Rluc-GND-transfected cells (Fig 3C). Depletion of Rab27a did not diminish translation of the input RNA (Fig 3B and 3C). However, translation of replicating RNAs was significantly decreased in Rab27a-depleted cells compared to control siRNA-treated cells. Importantly, the EMCV IRES activity was not affected by Rab27a depletion (S5 Fig), eliminating the possibility that these results were due to altered abundances of viral proteins. These findings argue that Rab27a plays a role in the viral life cycle by modulating HCV RNA replication.

**Fig 4:**
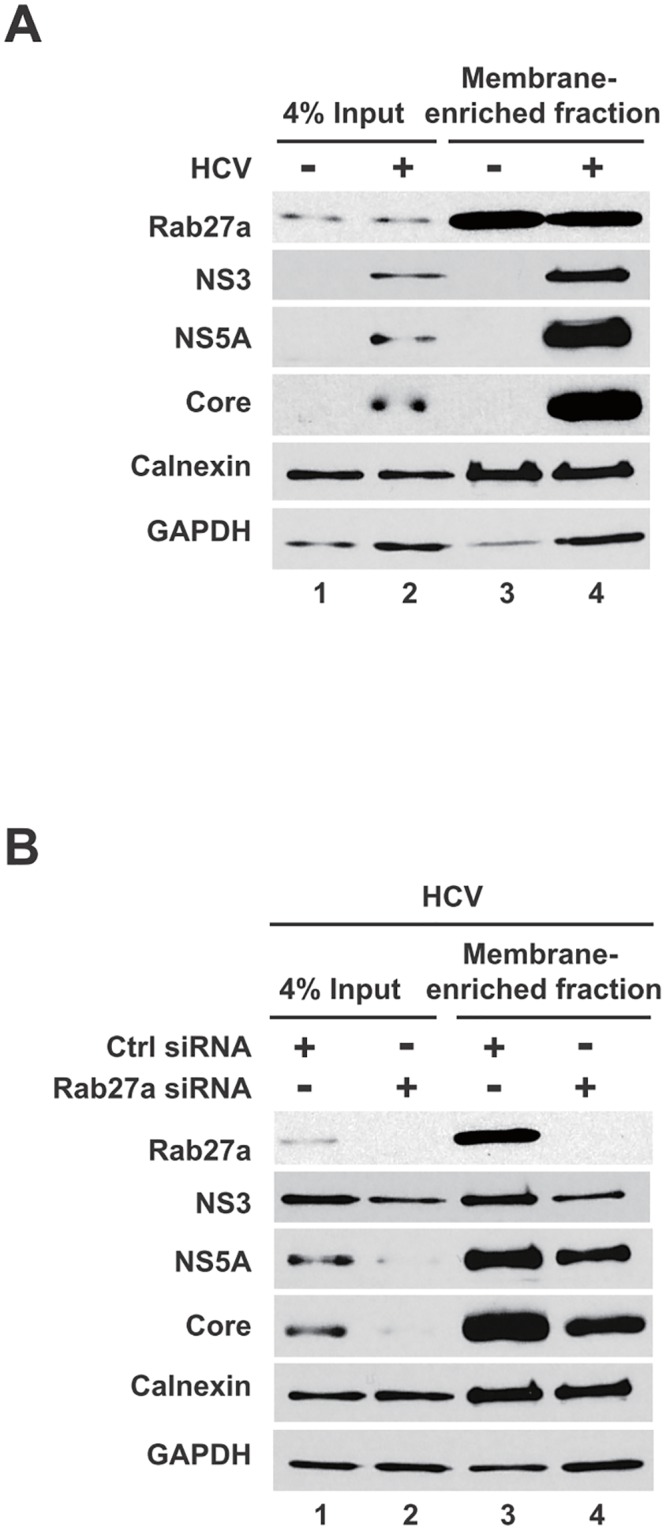
Presence of Rab27a and viral proteins membrane-enriched fractions. (A) Rab27a is present in membrane-enriched fractions. Lysates from uninfected- and HCV-infected cells were prepared in hypotonic buffer, and separated in a discontinue gradient. Proteins present in the enriched membrane fraction were extracted and analyzed by Western blot. (B) Rab27a depletion decreased viral protein abundance in membrane-enriched fractions. Samples from control- and Rab27a siRNA-transfected cells at day 3 post-infection with HCV were prepared and analyzed as described A.

It is known that cells expressing HCV replicons or cells that are infected with HCV display membrane rearrangements and formation of virus-induced membranous webs [11, 26–29]. The HCV-induced membranous webs, which are thought to be the sites of viral replication, are mainly derived from the endoplasmic reticulum (ER)[29]. To examine whether Rab27a is located to membranes during HCV RNA replication, membrane-enriched fractions from uninfected and HCV-infected cells were isolated, using discontinuous sucrose gradients. Western blot analyses showed that the membrane fractions contained the ER membrane marker protein calnexin (Fig 4A and 4B, lanes 3 and 4). In addition, HCV proteins NS5A, NS3 and capsid protein core also located to these fractions (Fig 4A and 4B). Interestingly, Rab27a was also found to localize in the membrane-enriched fraction. Rab27a depletion caused a decrease of HCV NS3, NS5A and core protein abundance in the enriched-membrane fraction (Fig 4B, lane 4), but not calnexin or GAPDH. These results indicate that Rab27a is associated with membrane-enriched fractions in infected cells, and that Rab27a depletion selectively diminished the abundance of several viral non-structural proteins in the replication complex-containing membranes.

**Fig 5:**
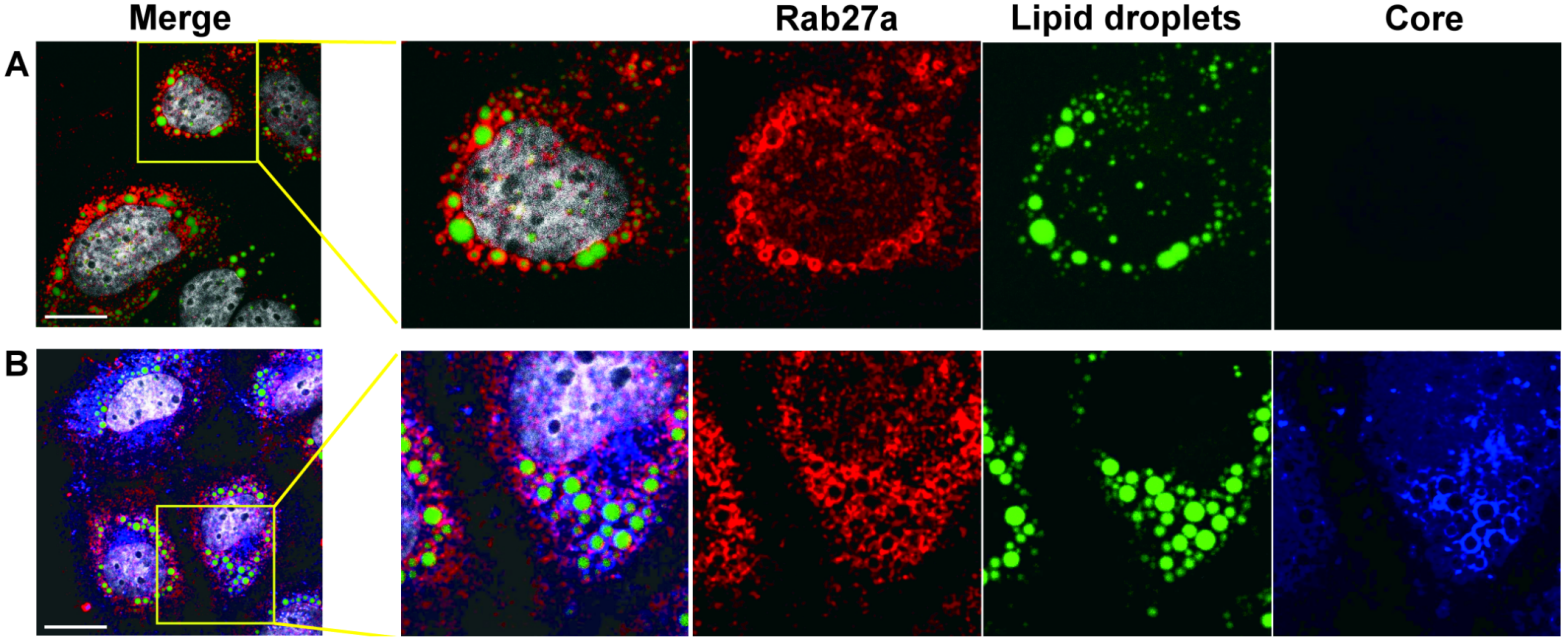
Subcellular localization of Rab27a. (A) Subcellular localization of Rab27a and lipid droplet in uninfected Huh7 cells. (B) Subcellular localization of Rab27a (red), HCV core (blue) and lipid droplets in HCV-infected cells at day 3 post-infection. Lipid droplets were stained with Bodipy 493/503 (green) and nuclei were stained with Hoechst 33258 (white). Scale bar, 20 μm.

The above genetic and biochemical findings argue that Rab27a regulates HCV RNA replication via its association with virus-induced membranes. To further substantiate this hypothesis, the subcellular location of Rab27a was investigated by confocal immunofluorescence microscopy. Astonishingly, Rab27a exhibited a doughnut-like structural localization around lipid droplets (LDs) (Fig 5 and S6 Fig) in uninfected (Fig 5A) and in infected liver cells (Fig 5B). These findings suggest that Rab27a may have a hitherto unknown role in the metabolism of LDs in liver cells. The LD-Rab27a doughnut-like structures colocalized with viral core protein in infected cells (Fig 5B). In addition, a small fraction of NS3 displayed a punctate distribution in the LD-Rab27a structures, indicating that Rab27a localizes to adjacent to sites of viral replication (S6B Fig).

### Effect of Rab27a depletion on HCV RNA stability

**Fig 6:**
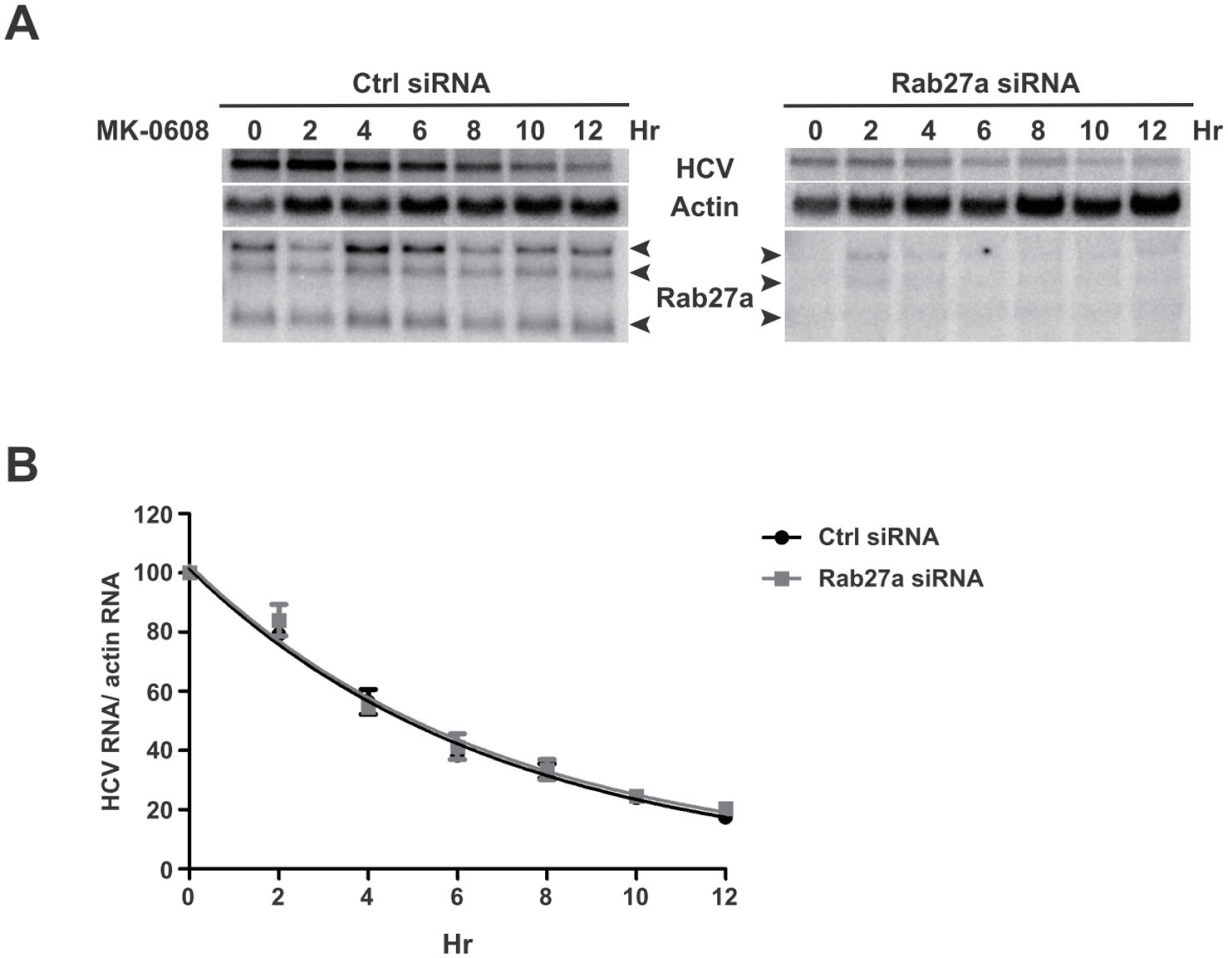
HCV RNA stability in Rab27a-depleted cells. (A) Effects of Rab27a on HCV RNA decay. Cells were transfected with control or Rab27a siRNAs one day prior to electroporation of HCV RNA. After 3 days post-electroporation, the cells were treated with 25 μM of MK0608, and RNAs were extracted every two hours for 12 hours. HCV RNA abundance was measured by Northern blot. (B) One phase decay graph of HCV RNA. The normalized HCV RNA amounts were determined by Northern blot in three independent experiments. Data were fit to a one-phase decay model (R^2^ = 0.96–0.98). Estimated half-life (t_1/2_) of HCV RNA is 4.8 hours in both control- and Rab27a-depleted cells.

The impaired HCV gene and protein expression may be due to a lack of stabilization of HCV RNA. To examine whether Rab27a affects HCV RNA stability, Huh7 cells were transfected with control- or Rab27a-siRNAs, followed by addition of the NS5B inhibitor MK-0608 to block new synthesis of HCV RNA. The rate of HCV RNA decay was determined by Northern blot analysis at different times after addition of MK-0608 (Fig 6A). Viral RNAs from control- and Rab27a-depleted samples displayed similar decay rates, with approximate half-lives of 4.8 hours (Fig 6B). These results indicate that Rab27a depletion affects the rate of HCV RNA replication without changing HCV RNA stability.

### Rab27a-depletion selectively decreases miR-122 abundance in cells

**Fig 7:**
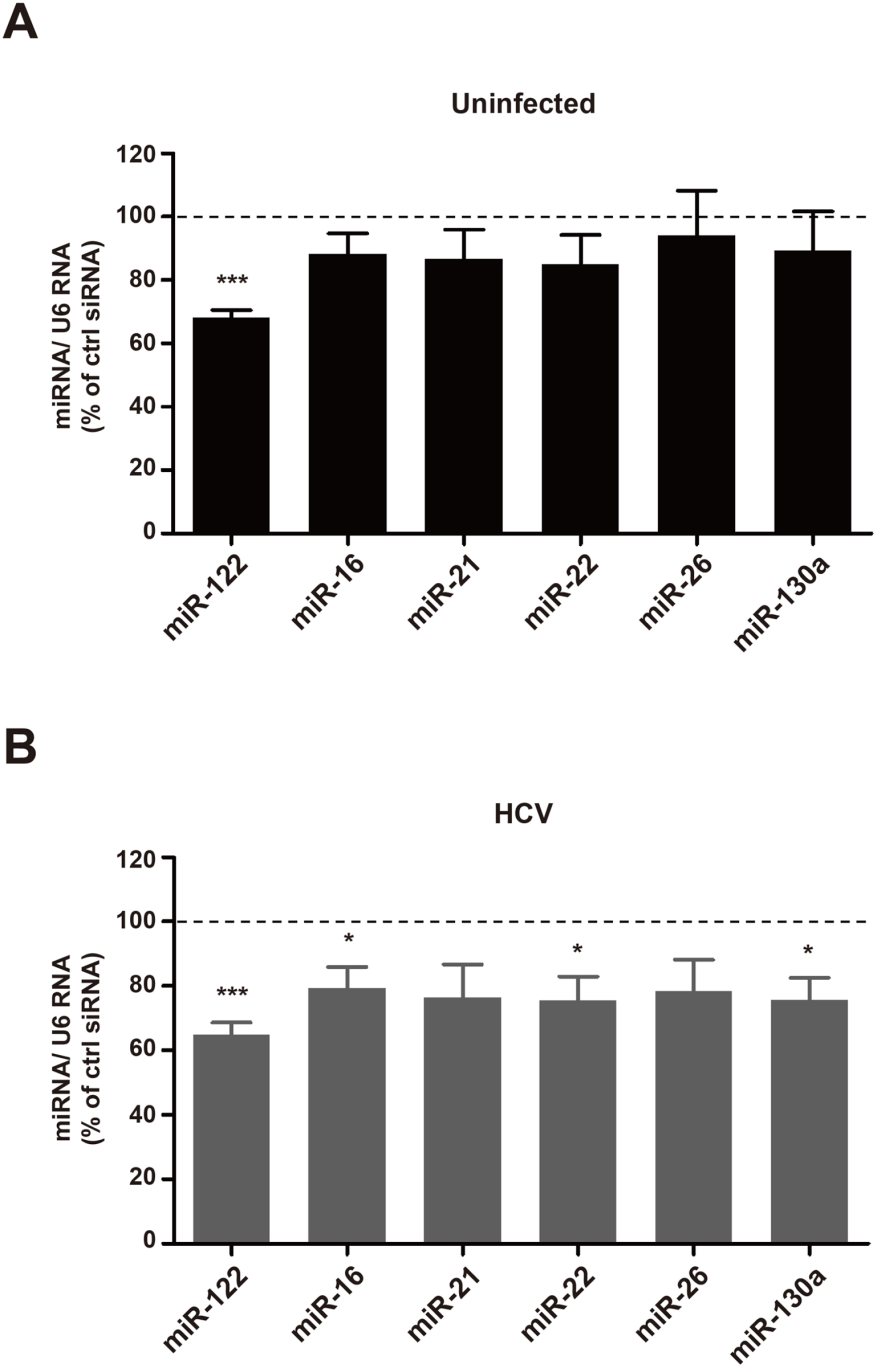
Effects of Rab27a depletion on microRNA abundance. Abundance of miR-122, miR-16, miR-21, miR-22, miR-26a and miR-130a, in control- or Rab27a-depleted cells were measured by small RNA Northern blot analysis. MiRNA abundances were normalized to that of U6 snRNA. Relative miRNA abundance in Rab27a-depleted cells in uninfected (A) and HCV-infected (B) cells. MiRNA abundance in cells treated with control siRNA is set to 100%. The data are representative of six independent replicates (*P<0.05 and ***P<0.0005, Student’s t-test).

It is known that miR-122 modulates HCV RNA expression [3, 30]. Therefore, it is possible that the observed effects of Rab27a depletion on the rates of HCV RNA replication could be due to altered abundance of miR-122. Thus, intracellular miR-122 abundance was monitored in Rab27a-depleted cells by Northern blot analysis. Results showed that miR-122 abundance was decreased by more than 30% in both uninfected- and HCV-infected Rab27a-depleted cells (Fig 7A and 7B). This was surprising because miR-122 has been reported to be quite stable in liver cells [31]. A luciferase reporter-based assay also showed diminished miR-122 function in Rab27a-depleted cells (S7 Fig). While the abundances of five other endogenous miRNAs (miR-16, miR-21, miR-22, miR-26 and miR-130a) were not changed in uninfected, Rab27a-depleted cells (Fig 7A), the abundances of miR-16, miR-22 and miR-130a showed a modest decrease in Rab27a-depleted cells during HCV infection; but not to the same extent as miR-122 (Fig 7B).

**Fig 8:**
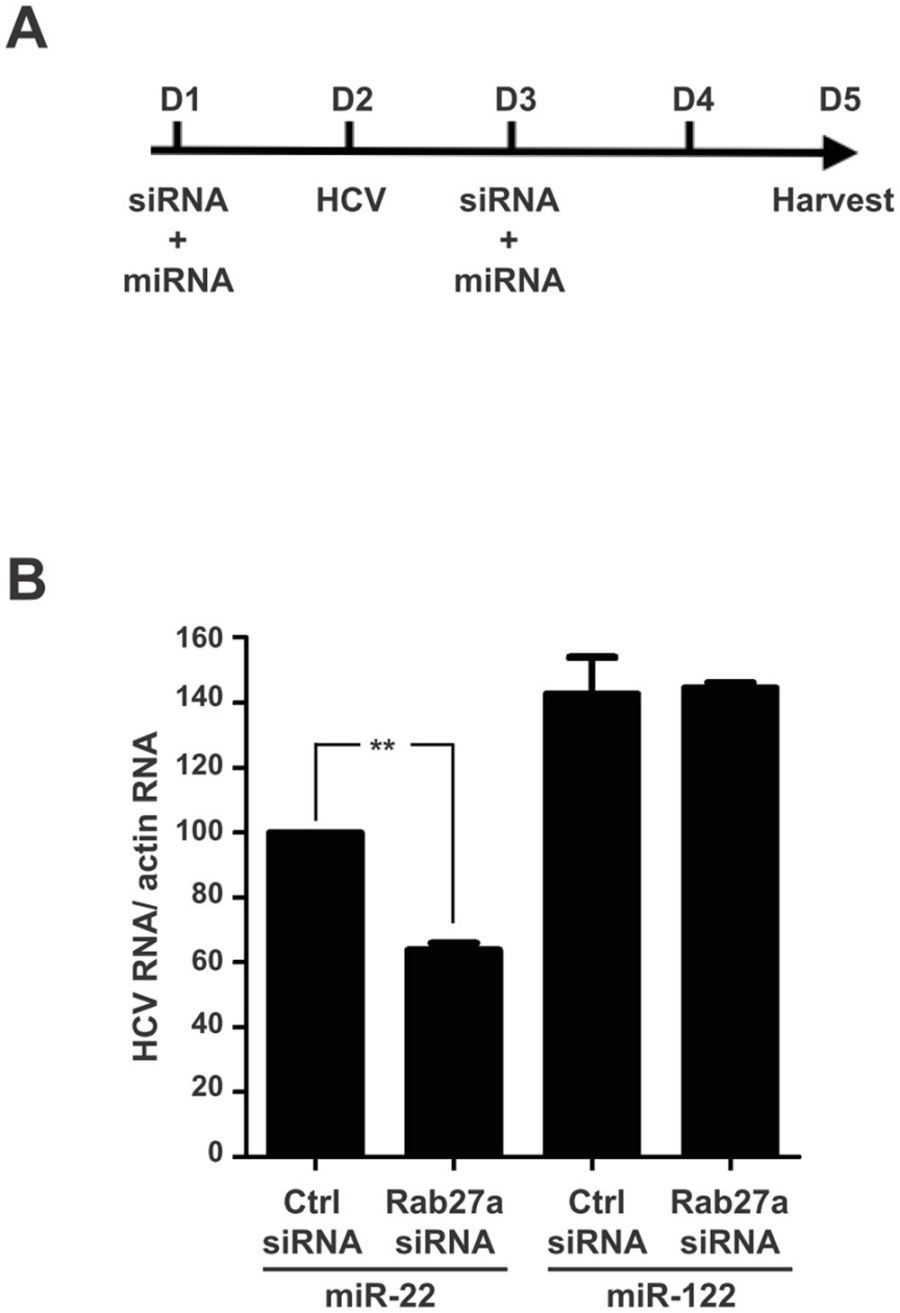
Rescue of HCV RNA in Rab27a-depleted cells by ectopic expression of miR-122. (A) Diagram of experimental design. Huh7 cells were co-transfected with Rab27a siRNA or control siRNA and miR-122 or miR-22 one day before and after virus infection. (B) Overexpression of miR-122 rescues the effect of Rab27a depletion on HCV RNA abundance. HCV RNA abundance during Rab27a depletion and miR-122 overexpression was measured by Northern blot and normalized to actin mRNA. Relative HCV RNA abundance in cells transfected with control siRNA and miR-22 is set to 100%. The data are from four independent replicates (**P<0.005, Student’s t-test).

To test whether the modulation of HCV RNA replication by Rab27a was caused by the altered abundance of miR-122 or any other microRNA, we investigated whether miR-122 overexpression prevented the Rab27a-dependent inhibition of HCV RNA replication (Fig 8A). Fig 8B shows that overexpression of miR-122 mimetics could rescue HCV RNA abundance in Rab27a-depleted cells, while the overexpression of miR-22 had no effects. A similar result was observed during overexpression of miR-21 as a control. These findings suggest that the decrease of HCV RNA abundance in Rab27a-depleted cells is due to the reduction in miR-122 abundance and is not due to the reduction of other microRNAs, such as miR-22 (Fig 8B).

We next examined whether Rab27a modulates the transcription of miR-122. Primary miR-122 (pri-miR-122) transcript abundance was examined in Rab27a-depleted uninfected or HCV-infected cells. S8A and S8B Fig shows that the abundance of pri-miR-122 is not affected by the depletion of Rab27a in uninfected and infected cells, suggesting that Rab27a modulates miR-122 abundance at a post-transcriptional step. Because precursor-miR-122 (pre-miR-122) can not be detected in cultured Huh7 cells, we determined the effect of Rab27a on the stability of a pre-miR-122 species that is resistant to the cleavage by Dicer [32]. Thus, the intracellular decay of a dicer-resistant pre-p3 (dNx12) that is functional in regulating mRNAs with miR-122 target sites [32] was examined (S9A Fig). Control- or Rab27a-siRNA treated cells were transfected with 5’-^32^P-labelled pre-p3 (dNx12) mimetics and the abundance of the labeled pre-miRNAs was determined at one day after transfection. The three independent experiments in S9B Fig show that the abundance of 5’-^32^P-labelled pre-p3 (dNx12) significantly decreased by the depletion of Rab27a (S9C Fig), arguing that Rab27a likely diminished miR-122 abundance by decreasing pre-miR-122 abundance.

### Effect of Rab27a-depletion is independent of the HCV 5’ UTR-miR-122 interaction

**Fig 9:**
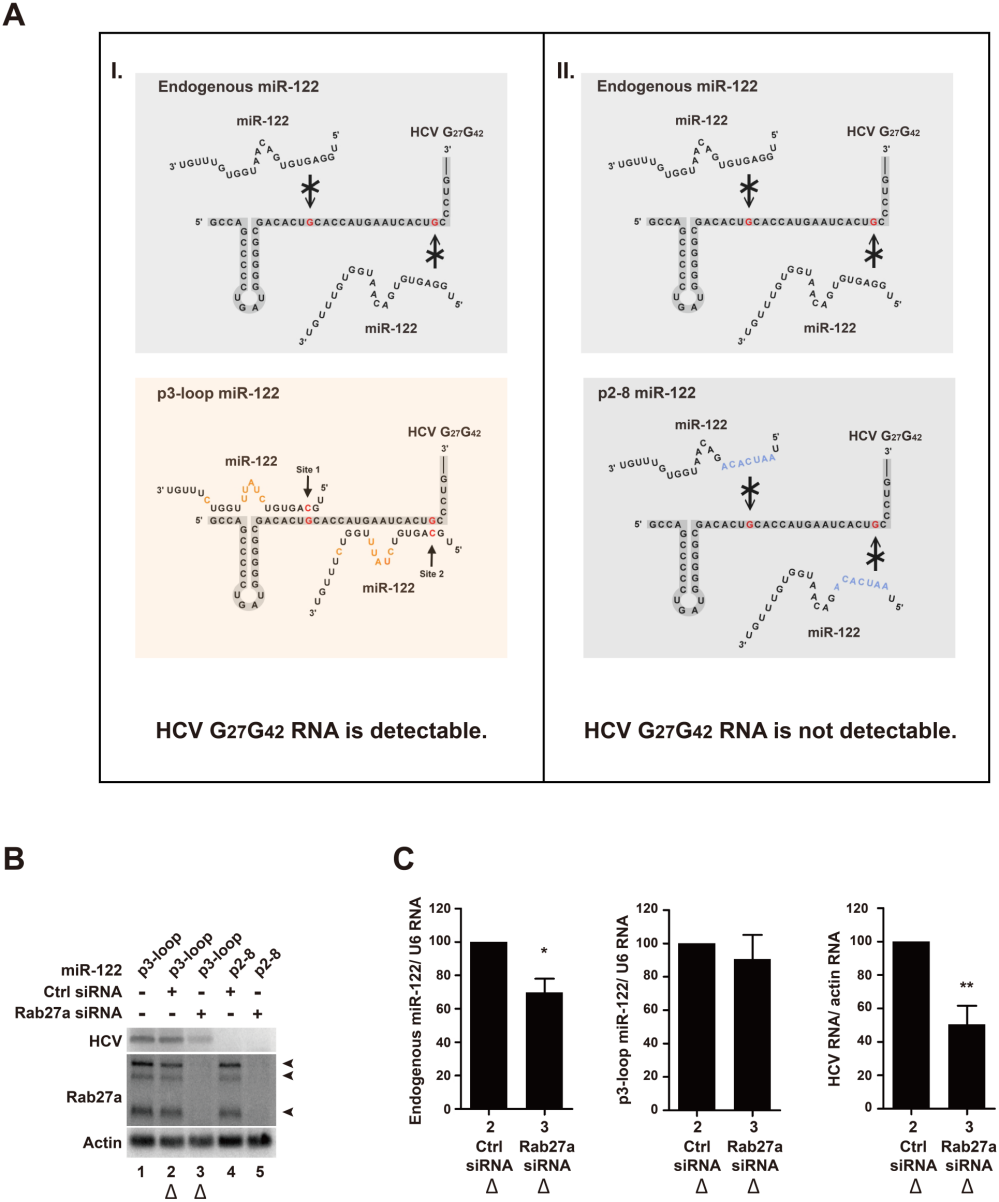
Effects of Rab27a depletion on HCV RNA abundance are independent of the 5’ terminal miR-122:HCV complex. (A) I, Diagram representing interactions between endogenous wildtype (top) and mutant, p3-loop miR-122 molecules (bottom) with a mutant HCV RNA genome (HCV G_27_G_42_). Mutations in the HCV G_27_G_42_ RNA genome are highlighted in red. A complementary mutation in miR-122 at position 3 is highlighted in red (p3-loop). Additional mutations in p3-loop mimetics at position 9–13 and 18 are highlighted in orange (bottom). II, Diagram representing interactions between endogenous wildtype (top) and p2-8 miR-122 molecules (bottom) with a HCV G_27_G_42_ RNA genome. Seed mutations in mutation p2-8 are highlighted in blue. (B) Accumulation of HCV, Rab27a and actin RNA transcripts. Cells were transfected with control or Rab27a siRNAs at day 1, transfected with the p3-loop or p2-8 miR-122 at day 2, and electroporated with the HCV G_27_G_42_ RNA at day 3. The cells were transfected again with siRNAs and miR-122 duplexes at day 4. Cells were harvested at day 3 post-electroporation, and RNAs were measured by Northern blot analysis. (C) Quantitation of normalized HCV G_27_G_42_ RNA, p3-loop miR-122 and endogenous miR-122 abundances in Rab27a-depleted cells of samples shown in Fig 9B, lane 2 and 3 (marked with triangles). Data in control siRNA treated cells was set as 100%. The data are representative of five independent replicates (*P<0.05 and **P<0.01, Student’s t-test).

It is known that two miR-122 molecules protect the 5’-terminal sequence of the HCV RNA genome from exonucleolytic degradation [5, 6]. Thus, it was possible that the reduced level of intracellular miR-122, after Rab27a depletion, caused the decrease in HCV RNA abundance by leaving the viral RNA unprotected. To test this possibility, a mutant HCV RNA genome (HCV-G_27_G_42_) that contained a mutation at each of the two miR-122 binding sites at the 5’ UTR was generated (Fig 9A, nucleotides highlighted in red). When transfected into cells, HCV-G_27_G_42_RNA cannot replicate because it cannot bind endogenous miR-122 (Fig 9A, (I), left upper panel) [3, 4, 30]. However, introduction of p3-loop miR-122 molecules that harbor a compensatory mutation at position 3 (red), and additional mutations at positions 9–13 (orange) and 18 (orange) (Fig 9A, (I), lower panel) can enhance HCV-G_27_G_42_ RNA abundance (Fig 9B, lanes 1 and 2). The nucleotide changes 9–13 (orange) and 18 (orange) in p3-loop miR-122 allow us to distinguish p3-loop miR-122 from endogenous wildtype miR-122 in Northern blots. As a negative control, miR-122 molecules with mutations in their entire seed sequences (p2-8; nucleotides highlighted in blue) (Fig 9A, (II), lower panel) did not enhance HCV-G_27_G_42_ RNA abundance (Fig 9B, lane 4). This finding shows that the HCV-G_27_G_42_ RNA genome abundance was enhanced by p3-loop miR-122, and not by endogenous miR-122 or p2-8 miR-122. Expression of p3-loop miR-122 mimetics allowed a 50% of HCV RNA accumulation in Rab27a-depleted cells (Fig 9B, lane 3) compared to cells that were not depleted of Rab27a (Fig 9B, lanes 1 and 2). Quantitation of the abundances of the endogenous and p3-loop miR-122 molecules revealed that endogenous miR-122 abundance was diminished by 30% in Rab27a-depleted cell (Fig 9C), a finding that is consistent with the result in Fig 7A and 7B. In contrast, the abundance of p3-loop miR-122 was not affected by Rab27a depletion (Fig 9C). Therefore, the 50% decrease in HCV-G_27_G_42_ RNA abundance in Rab27a-depleted cells in the presence of p3-loop miR-122 mimetics (Fig 9B, lane 3), is independent of the interaction of p3-loop miR-122 with the 5’ end of HCV RNA. This findings argue that endogenous miR-122, but not p3-loop miR-122, downregulates the expression of an inhibitor of HCV RNA gene expression.

## Discussion

CD81-containing exosomes are multivesicular body-derived microvesicles found in eukaryotic cells and are involved in cell-to-cell communication. It has been shown that both mRNAs and miRNAs can be transferred into neighboring cells by this pathway [33], and that HCV RNA can also be secreted from infected cells by extracellular vesicles [12, 14, 21–23]. However, extracellular vesicles, including exosomes, can be derived from several distinct pathways. To test whether HCV RNA and miR-122 are secreted by bona-fide exosomes, Rab27a that modulates the docking of multivesicular bodies to the plasma membrane [16] was depleted by siRNAs. Indeed, depletion of Rab27a led to a decrease of CD81- and CD63-positive exosome secretion in Huh7 cells (S1 Fig). Previous studies have argued that viral RNA can be transferred by “exosomes” [12, 14, 21–23], which were isolated from supernatants of cultured cells by subsequent centrifugation steps and CD81 affinity chromatography. In contrast, we show here that depletion of exosomes by genetic downregulation of the exosome docking protein Rab27a lowered both the intracellular and extracellular abundance of HCV RNA and virions (Fig 2), arguing that microvesicles other than exosomes are the major vehicles for the transport of viral RNA and virions.

The effect of Rab27a siRNA-3, which targets the 3’ noncoding region of all Rab27a mRNAs, on HCV RNA abundance could not be restored by overexpressing a knockdown-resistant Rab27a variant. We also found that overexpression of Rab27a did not increase HCV RNA and extracellular exosome abundance. Thus, Rab27a may affect HCV RNA abundance and exosome secretion as part of a protein complex. Alternatively, siRNA-3 caused off-target effects that were unrelated to Rab27a. To examine the latter possibility, additional siRNAs targeting different regions of Rab27a mRNAs were tested. All siRNAs showed a decrease in HCV RNA abundance, supporting the specificity of Rab27a’s effect on HCV RNA abundance (S3 Fig). Importantly, all siRNAs directed against Rab27a did not affect cell viability.

Studies with HCV replicons provided genetic evidence that Rab27a modulates the rate of viral replication (Fig 3). To further substantiate this finding with a biochemical approach, we examined the protein composition of membranes, which are sites for viral RNA replication. A substantial amount of Rab27a located to membrane-enriched fractions, both in uninfected and infected cells (Fig 4). In addition, confocal microscopy studies revealed that Rab27a localizes to LDs in uninfected and infected cells (Fig 5). Curiously, Rab27a coats LDs, visualizing the Rab27a-LDs complex as a doughnut-shaped structure. LD-associated Rab27a colocalized with viral core protein and with a small fraction of NS3. It has been proposed that HCV core recruits ER-derived membrane webs that are close to LDs to create a local membrane environment for viral replication and assembly [34, 35]. While the exact mechanism by which Rab27a modulates HCV RNA abundance is not clear at present, our findings strongly argue that Rab27a regulates HCV RNA abundance at LDs.

It is known that the presence of miR-122 is essential to maintain HCV RNA abundance. Profiling of several microRNAs in Rab27a-depleted cells showed that the abundance of miR-122 was decreased both in uninfected and infected cells. The loss of HCV RNA abundance during Rab27a depletion could be rescued by overexpression of miR-122 mimetics, which is consistent with the hypothesis that Rab27a-mediated depletion of miR-122 caused loss of HCV RNA abundance (Fig 8).

No significant decrease in the amount of primary miR-122 was observed in Rab27a-depleted cells, indicating that the effect of Rab27a depletion on miR-122 most likely occurred at a post-transcriptional step in the cytosol. Indeed, Rab27a depletion caused a decrease of ectopically expressed precursor miR122 (S9 Fig). It has been reported that both pre-microRNAs and mature microRNAs can be released from cells via exosomes that contain the GW182 component of the RNA-induced silencing complex (RISC) [36, 37]. This observation raises the possibility that depletion of Rab27a enhances the intracellular abundance of GW182-containing vesicles that affect the stability of pre-miR122 or miRNA-122 molecules. However, depletion of Rab27a effector Slp4 did not affect miR-122 and HCV RNA abundances. Because depletion of Slp4 inhibits exosome trafficking [16], loss of HCV RNA and miR-122 was not due to the accumulation of intracellular exosomes. We hypothesize that pre-miR-122 is being destabilized in the absence of Rab27a by an as-of-yet unknown mechanism. We also noted a selective decrease of several microRNAs in infected cells. One explanation is that HCV infection causes a dispersion of Processing bodies, where microRNAs, microRNA-targeted mRNAs and Argonaute proteins are located [38, 39]. This dispersion may affect turnover of specific microRNAs in infected cells. Alternatively, HCV is known to sequester components of RISC, such as Ago2 and GW182, at the HCV 5’ end for maintaining viral genome stability [40]. As a consequence, RISC-free microRNAs may be more easily degraded [22, 41, 42]. It is important to note that both miR-122 and miR-22 are depleted in HCV-infected cells. However, only the depletion of miR-122 affects HCV RNA abundance (Fig 8), arguing that loss of HCV RNA abundance was not caused by an overall loss of microRNAs in infected cells.

We examined whether loss of miR-122 led to the accumulation of HCV-G_27_G_42_ RNA molecules that were vulnerable to exonuclease cleavage. Thus, we examined the abundance of HCV-G_27_G_42_ RNA that could be protected by ectopically expressed mutant miR-122 molecules in Rab27a-depleted cells. The abundance of HCV-G_27_G_42_ RNA that could interact with mutant miR-122, but not with endogenous miR-122, also decreased in Rab27a-depleted cells (Fig 9). Because mutant miR-122 molecules very likely do not recognize mRNA targets that are modulated by wildtype, endogenous miR-122, effects of endogenous miR-122 on HCV RNA abundance are by a mechanism that is different from its protecting the 5’ end of the viral RNA. We also examined whether a miR-122 antagonist, instead of Rab27a depletion, caused a decrease in miR-122 to affect HCV replication that is independent of endogenous miR-122. We noted to our surprise that exogenously expressed mutant miR-122 mimetics cannot be functionally sequestered by the employed antagomirs. Thus, it is possible that an antagomir-inaccessible pool of mutant miR-122 accumulates within the transfected cell.

Finally, depletion of Rab27a has no effect on exoribonucleases Xrn1 and Xrn2 abundance. Thus, it is very likely that miR-122 downregulates an inhibitor of HCV gene expression. Such an inhibitor is not involved in the biosynthesis of cholesterol, because cholesterol abundance is not affected in Rab27a-depleted uninfected or infected cells.

## Methods

### Cell culture

Human hepatoma Huh7 cells were kindly provided by Francis V. Chisari (The Scripps Research Institute, San Diego). Huh7 cells were cultured in DMEM supplemented with 10% fetal bovine serum, 1x non-essential amino acids and 2 mM L-glutamine (Gibco).

### Oligonucleotides

All Small interfering RNA (siRNA) oligonucleotides and other RNA oligonucleotides were synthesized by Stanford PAN facility (Stanford, CA). The siRNA sequences are as follow: siControl, 5’- GAUCAUACGUGCGAUCAGAdTdT-3’; siRab27a-1: 5’- GGAGAGGUUUCGUAGCUUAdTdT-3’; siRab27a-2: 5’- GCCUCUACGGAUCAGUUAAdTdT-3’. The RNA oligonucleotide sequences are as follow: p3-loop miR-122: 5’- UGCAGUGUCUAUUUGGUCUUUGU-3’; p2-8 miR-122: 5’- UAAUCACAGACAAUGGUGUUUGU-3’. For formation of RNA duplexes, 50 μM of sense and antisense strands were mixed in annealing buffer (150 mM HEPES (pH 7.4), 500 mM potassium acetate, and 10 mM magnesium acetate) to a final concentration of 20 μM, denatured for 1 min at 95°C, and annealed for 1 h at 37°C.

### Generation of HCV

Huh7 cells (10^6^) were seeded in 10 cm tissue culture dishes. Cells were infected with wild-type JFH1 at a MOI of 0.01 for 5 h, washed with PBS to remove unbound virus, trypsinized and replated in 15 cm tissue culture dishes. The supernatant was collected at 3 days post-infection and centrifuged at 1,000 rpm, 10 min at 4°C to remove cell debris. The infected cells were scraped and resuspended in medium and subjected to freezed-thraw cycles. Samples were centrifuged at 1,000 rpm, 10 min at 4°C to remove cell debris. For the virus stock, the supernatant was mixed with cell-associated virus. Virus was stored in aliquots at -80°C. Virus titter was determined by using fluorescent focus-forming assay.

### Small interfering RNA transfection and HCV infection

Huh7 cells (2.5 x 10^5^) were seeded in 60 mm tissue culture dishes. Cells were transfected the following day with 50 nM of siRNA duplexes (25nM siRab27a-1 plus 25 nM siRab27a-2) using Dharmafect I reagent (Dharmacon) according to the manufacturer’s instruction. After 24 h post-transfection, the cells were infected with HCV JFH-1 virus at a MOI of 0.01 at 37°C. After 5 h incubation, cells were washed with PBS to remove unbound virus, trypsinized and replated in duplicate tissue culture dishes. Virus-infected cells were transfected again with 50 nM of siRNA duplexes at day 1 post-infection, and harvested at day 3 post-infection. The efficiency of siRNA depletion was evaluated by Northern and Western blot analysis.

### RNA Isolation and Northern analysis

Huh7 cells were washed once with PBS and total RNA was extracted using TRIzol (Invitrogen) following the manufacturer’s protocol. Ten μg of total RNA in RNA loading buffer (32% formamide, 1x MOPS-EDTA-Sodium acetate (MESA, Sigma) and 4.4% formaldehyde) was denatured at 65°C for 10 min and separated in a 1% agarose gel containing 1x MESA and 3.7% formaldehyde. The RNA was transferred and UV crosslinked to a Zeta-probe membrane (Bio-Rad). The membrane was hybridized using the ExpressHyb hybridization buffer (Clontech) or ULTRAhyb (Ambion) and α-^32^P dATP-RadPrime DNA labelled probes (Invitrogen) complementary to HCV (nucleotides 84–374), Rab27a (nucleotides 664–1145), or actin (nucleotides 685–1171). Autoradiographs were quantified using ImageQuant (GE Healthcare).

### Small RNA Northern analysis

Ten μg of total RNA was separated in 12% acrylamide/ 7 M urea gel. Small RNAs were transferred onto a Hybond-N+ membrane (GE Healthcare), and detected by *γ*-^32^P-end labelled DNA probes complementary to miR-122, miR-16, miR-21, miR-22, miR-26, miR-130a, mutant miR-122 or U6 snRNA. Oligonucleotide sequence of probes are: miR-122 probe, 5’-CAAACACCATTGTCACACTCCA-3’; miR-16-5p probe, 5’-CGCCAATATTTACGTGCTGCTA-3’; miR-21 probe, 5’-TCAACATCAGTCTGATAAGCTA-3’; miR-22-3p probe, 5’-ACAGTTCTTCAACTGGCAGCTT-3’; miR-26a-5p probe, 5’- AGCCTATCCTGGATTACTTGAA-3’; miR-130a-3p probe, 5’- ATGCCCTTTTAACATTGCACTG-3’; U6 probe, 5’-CACGAATTTGCGTGTCATCCTTGC-3’. The membrane was hybridized using 7.5 x Denhardt’s solution, 5 x SSPE, 0.1% SDS, 0.05 mg/ml tRNA. Autoradiographs were quantified using ImageQuant (GE Healthcare).

### Western blot analysis

Cells were washed with PBS once and lysed in RIPA buffer (50mM Tris (pH8.0),150 mM NaCl, 0.5% sodium deoxycholate, 0.1% SDS, and 1% Triton X-100) containing Complete EDTA-free protease inhibitors (Roche) for 15 min on ice. The cell lysate was clarified by centrifugation at 14,000rpm for 5 min at 4°C. Forty μg of cell lysate was mixed with 2x SDS sample buffer (126 mM Tris HCl, 20% glycerol, 4% SDS and 10% β-mercaptoethanol, 0.005% bromophenol blue, pH 6.8), denatured at 90°C for 5 min and separated in a 10% SDS-polyacrylamide gel. Protein was transferred to a PVDF membrane (Millipore). The membrane was blocked with 5% non-fat milk in PBS-T and probed using primary antibody, followed by horse-radish peroxidase-conjugated secondary antibodies. The blot was developed using Pierce ECL Western Blot Substrate (Thermo Scientific) according to the manufacturer’s instructions, and exposed to Biomax Light Films. The following primary antibodies were used for western blot analysis: anti-Core (C7-50) (Abcam, ab2740), anti-Rab27a (Abnova), anti-GAPDH (Calbiochem CB1001).

### Fluorescent focus-forming assay

Infectious titers were determined by measuring fluorescent focus forming units (FFU) [43]. Rab27a depleted cells were infected with JFH-1 virus. For extracellular virus, supernatant of the infected cells was collected at day 3 post-infection. To harvest cell-associated virus, infected cells were washed with PBS three times, collected into a new tube, and resuspended in 500 μl DMEM. The cells were frozen and thawed three times. Both extracellular and cell-associated supernatants were sedimented at 14,000 rpm, 4°C for 5 min to remove cell debris. The viral titer was determined by FFU assay. Briefly, 3.2 x 10^4^ cells were seeded in a 48-well plate and incubated overnight. A serial dilution of virus stock was added to cells and incubated for 5 h at 37°C. The diluted virus supernatant was removed from cells. Cells were washed with PBS and replaced with fresh medium. At day 3 post-infection, infected cells were washed once with PBS and fixed with cold methanol/acetone (1:1). The level of HCV infection in the cells was analyzed by using a mouse monoclonal antibody direct against HCV core (Abcam) at 1:1000 dilution in 1% fish gelatin/PBS at 4°C overnight and an AlexFluor488- conjugated goat anti-mouse antibody (Invitrogen) at 1: 200 dilution at room temperature for 2 h. The fluorescent focus forming units were counted using a fluorescence microscope, and the viral titer was expressed as FFU per ml.

### Preparation and quantification of extracellular HCV RNA

Cell culture supernatants were collected from infected Huh7 cells. HCV RNAs from the supernatant were isolated using TRIzol LS reagent (Inviterogen) following the manufacturer’s protocol. HCV transcripts were quantified using SuperScript III Platinum SYBR Green One-Step qRT-PCR kit (Invitrogen). The reactions were performed using the CFX connect Real-Time system (BIO-RAD). HCV transcript levels were determined by comparison to standard curves derived from in vitro transcribed HCV RNA. The primer sequences for JFH1 were, Fwd, 5’-TCTGCGGAACCGGTGAGTA-3’; Rev, 5’-TCAGGCAGTACCACAAGGC-3’.

### Electroporation of HCV RNA

The plasmid H77ΔE1/p7, containing a deletion of structural proteins E1-E2-p7 [44] was transcribed using the T7 MEGAscript kit (Ambion), according to the manufacturer’s protocol. A mutant HCV RNA (nucleotide 27 and 42 C to G change) from H77ΔE1/p7-S1+2:p3 was transcribed as described [3, 4, 39]. Huh7 cells were transfected with Rab27a siRNAs (50 nM) at day 1 and mutant miR-122 duplex (50 nM) at day 2. Subsequently, cells were electroporated with the mutant HCV RNA at day 3. Briefly, Huh7 cells in 10cm dishes were trypsinized, washed with PBS once, and then washed with the Cytomix buffer, and suspended in the Cytomix buffer (120mM KCl, 0.15 M CaCl_2_, 10mM K_2_HPO_4_, 25 mM HEPES, 2 mM EDTA, 5 mM MgCl_2_, pH7.6), containing 10 μg HCV RNA. The cells were electroporated in 0.4 cm Biorad cuvette at 900V, 25 μF, and *∞* resistance, then incubated at room temperature for 10 min and seeded in a new 10cm dish. The cells were transfected again with Rab27a siRNAs and mutant miR-122 duplexes at 1 day after electroporation and harvested 3 days after electroporation.

### HCV replication assay

Subgenomic JFH1-Rluc and JFH1-Rluc-GND were kindly provided by Glenn Randall (University of Chicago). The replicon RNA was generated using the T7 MEGAscript kit (Ambion) according to manufacturer’s protocols. Huh7 cells in 6 well plates were transfected with control or Rab27a siRNA using the Dharmafect I reagent (GE Dharmarcon). After 1 day post-transfection, cells were transfected with 2 μg of replicon RNA in TransMessenger reagent (Qiagen) for 1 h, and replaced with complete medium according to manufacturer’s instructions. Cells were harvested at 1, 2, 4, 8, 12, 24, 36 and 48 hours. Luciferase activity from the sample was detected according to manufacturer’s instructions.

### Isolation of enriched membranes

Membrane-enriched fractions were isolated using a modified protocol adapted from Schlegel et al. [45]. Briefly, cells were washed with cold PBS twice, scraped in PBS, and pelleted. Cells were suspended in hypotonic buffer (10 mM Tris (pH 8.0), 10 mM NaCl, 1 mM MgCl_2_, with complete protease inhibitor cocktail tablets (Roche) and 0.5 mM PMSF) for 10 min on ice and then homogenized for 50 strokes using a Dounuce homogenizer. The cell homogenate was centrifuged at 1,000 x g for 10 min to remove nuclei and unbroken cells. The supernatant was collected and salt concentration was adjusted by adding NaCl to a final concentration of 300 mM. The cytoplasmic extract was then layered on a 10% and 60% sucrose in 300 mM NaCl, 15 mM Tris-HCl (pH7.5), 15 mM MgCl_2_, and centrifuged at 26,000 rpm at 4°C in a SW41 rotor for 16 h. The viscous layer in the middle of the gradient was collected using a syringe. The sample was concentrated with a Nanosep 3K Omega centrifugal device (Pall Life Sciences). The sample was resuspended in 4 x SDS sample buffer and separated in a 10% SDS-polyacrylamide gel.

### Immunofluorescence staining

Uninfected and HCV-infected Huh7 cells were grown on 8-chambered coverglass slides (LabTek II chamber slides, Thermo Scientific) for 3 days. Cells were rinsed with PBS and fixed with 4% paraformaldehyde (Electron Microscopy Sciences) in PBS for 20 min at RT. Cells were then washed with PBS for 5 min twice, and permeabilized with 0.1% Triton X-100 in 1% fish gelatin (Sigma) in PBS (1% PBS-FG) for 5 min. Blocking incubation was performed in 1% PBS-FG for 10 min, 3 times, at RT. Cells were incubated with primary antibodies in 1% PBS-FG at 4°C overnight, washed with 1% PBS-FG for 10 min twice, and incubated with secondary antibodies for 2h at RT. To visualize lipid droplets, cells were by stained with BODIPY 493/503. After washing with 1% PBS-FG for 10 min twice, Hoechst 33258 dye (Sigma) in 1% PBS-FG was added and cells were incubated 5 min at RT. After two washes in 1% PBS-FG for 5 min each, the coverglass slides were embedded in Fluoromount-G (SouthernBiotech). Samples were imaged at RT (22°C) with a 20×/N.A.0.60 or a 63×/N.A.1.30 oil Plan-Apochromat objective on a Leica SPE laser scanning confocal microscope (Leica-microsystems). Images were processed with ImageJ (Ver. 1.48, NIH) using only linear adjustments of contrast and color.

The following antibodies and reagents were used for immunofluorescence staining. Primary antibodies: mouse anti-Rab27a (H00005873-M02, Abnova), goat anti-HCV core (2861, Virostat), goat anti-HCV NS3 (2871, Virostat). Secondary antibodies: Alex Fluor 555 conjugated donkey anti-goat IgG (H+L) (A-21432) and Alex Fluor 647 donkey anti-mouse IgG (H+L) (A-31571, Life technologies). Primary antibodies were used at 1:100 dilution and secondary antibodies were used at 1:200 dilution. Bodipy 493/503 was 1:100 dilution from 1 mg/ml stock (D3922, Invitrogen). Hoechst 33258 dye was 1:10,000 dilution from 2 mg/ml stock (Sigma).

### Statistical analysis

Statistical analyses were performed with Prism 5 (GraphPad). A two-tailed paired Student’s t-test was employed to assess significant differences between two groups. Error bars represent standard error of the mean.

## Supporting Information

S1 Fig Effect of Rab27a depletion on the amount of secreted CD81-containing exosomes. Control and Rab27a siRNA-treated cells were uninfected- (A) or HCV-infected (B). Supernatant was collected at day 3 post-infection from the cell culture medium and subjected to differential centrifugation (see S1 Methods). The resulting pellet (exosome fraction) and cell lysates were analyzed under non-reducing conditions by Western blot for Rab27a, CD81 and Calnexin. Quantification of CD81 protein abundance (right). The data are representative of five independent replicates (*P<0.05, Student’s t-test).
(TIF)

S2 Fig Effects of Rab27a depletion on cells infected with a high multiplicity of infection (MOI) with HCV. Cells were transfected with siRNAs at day 1 and infected with HCV at MOI = 10 at day 2. Cells were harvested 24 h post-infection. Effects on HCV RNA (A) and protein (B) abundance are shown in Northern and Western blot analyses, respectively.
(TIF)

S3 Fig Effect of Rab27a siRNAs on HCV RNA and protein abundance. (A) Northern blot analysis of HCV and Rab27a mRNA abundance. Data is representative of at least three independent experiments. (B) Western blot analysis of Rab27a and HCV Core. GAPDH served as a loading control. Immunoblot is representative of three independent experiments.
(TIF)

S4 Fig Effect of Rab27a siRNAs on cell viability (A) and apoptosis (B). (A) MTT assay of Rab27a siRNA-transfected cells. Control siRNA transfected cells was set to 100%. Cell death siRNA was used as a control for cell viability. The data are representative of four independent experiments (**P<0.005, Student’s t-test). (B) Control or Rab27a siRNA-treated cells were infected with HCV and harvested at day 3 post-infection. Apoptosis induction was assessed by PARP cleavage. Lysate from cells treated with cycloheximide (CHX) at 10 μg/ml and TNF-α at 50 ng/ml for 18 hr was used as a positive control. β-Actin served as loading control. Immunoblot is representative of three independent experiments.
(TIF)

S5 Fig Effect of Rab27a depletion on EMCV IRES activity. Huh7 cells were transfected with control or Rab27a siRNAs at 50 nM one day prior to pRL-EMCV IRES-FF plasmid transfection. Activities of firefly and Renilla luciferase were measured 24 hours later. The EMCV IRES activity (ratio of firefly luciferase to Renilla luciferase) in control siRNA-transfected cells was set to 100%. The data are representative of three independent experiments.
(TIF)

S6 Fig The distributions of Rab27a and HCV NS3 in uninfected and infected cells. Huh7 cells were uninfected (A) or HCV-infected (B) and then immune-stained for endogenous Rab27a (red) and NS3 (blue). Lipid droplets were stained with Bodipy 493/503 (green) and nuclei were stained with Hoechst 33258 (white). Scale bar, 20 μm.
(TIF)

S7 Fig miR122 activity in Rab27a-depleted cells. miR-122 activity was determined in control and Rab27a-depleted cells expressing plasmid pLUC-122x2 that transcribes firefly luciferase mRNA which contains miR-122 binding sites in its 3’ noncoding region. The cells were co-transfected with a Renilla reporter plasmid as a transfection control (see S1 Methods). The data are representative of three independent replicates (*P<0.05, Student’s t-test).
(TIF)

S8 Fig Effect of Rab27a depletion on pri-miR-122. (A) Effect on pri-miR-122 abundance. Control or Rab27a siRNAs-treated cells were uninfected- or HCV-infected. The abundance of pri-miR-122 was measured by Northern blot analysis 3 days post-infection. (B) Quantification of pri-miR-122. Pri-miR-122 was normalized to actin mRNA. Data from control siRNA treated cells was set to 100%. The data are representative of four independent replicates.
(TIF)

S9 Fig Effect of Rab27a on pre-miR-122 stability. (A) Sequence and predicted structure of Dicer-resistant pre-p3 miR-122(dNx12). The deoxynucleotides are highlighted in a box. The mutated C-nucleotide at position 3 in mature miR-122 is underlined. (B) Effect on pre-p3 miR-122(dNx12). Control and Rab27a siRNA-treated cells were transfected with 5’-^32^P-labelled pre-p3-miR-122(dNx12). Cells were harvested one day post-transfection. The total RNA containing 5’-^32^P-labelled pre-p3 miR-122(dNx12) was separated by gel electrophoresis, transferred onto a Hybond-N+ membrane. Autoradiograph of membranes from three independent experiments are shown. To generate a loading control, the membranes were subsequently hybridized with a labelled DNA probe that is complementary to U6 snRNA. Three independent experiments are shown in (B) and quantitated in (C).
(TIF)

S1 Methods Supplementary Methods. (DOCX)
