# Supplementary figures and images for: Supporting Role for GTPase Rab27a in Hepatitis C Virus RNA Replication through a Novel miR-122-Mediated Effect
Source: PLoS Pathog. 2015 Aug 25;11(8):e1005116. doi: 10.1371/journal.ppat.1005116 (PMC4549268; doi:10.1371/journal.ppat.1005116)

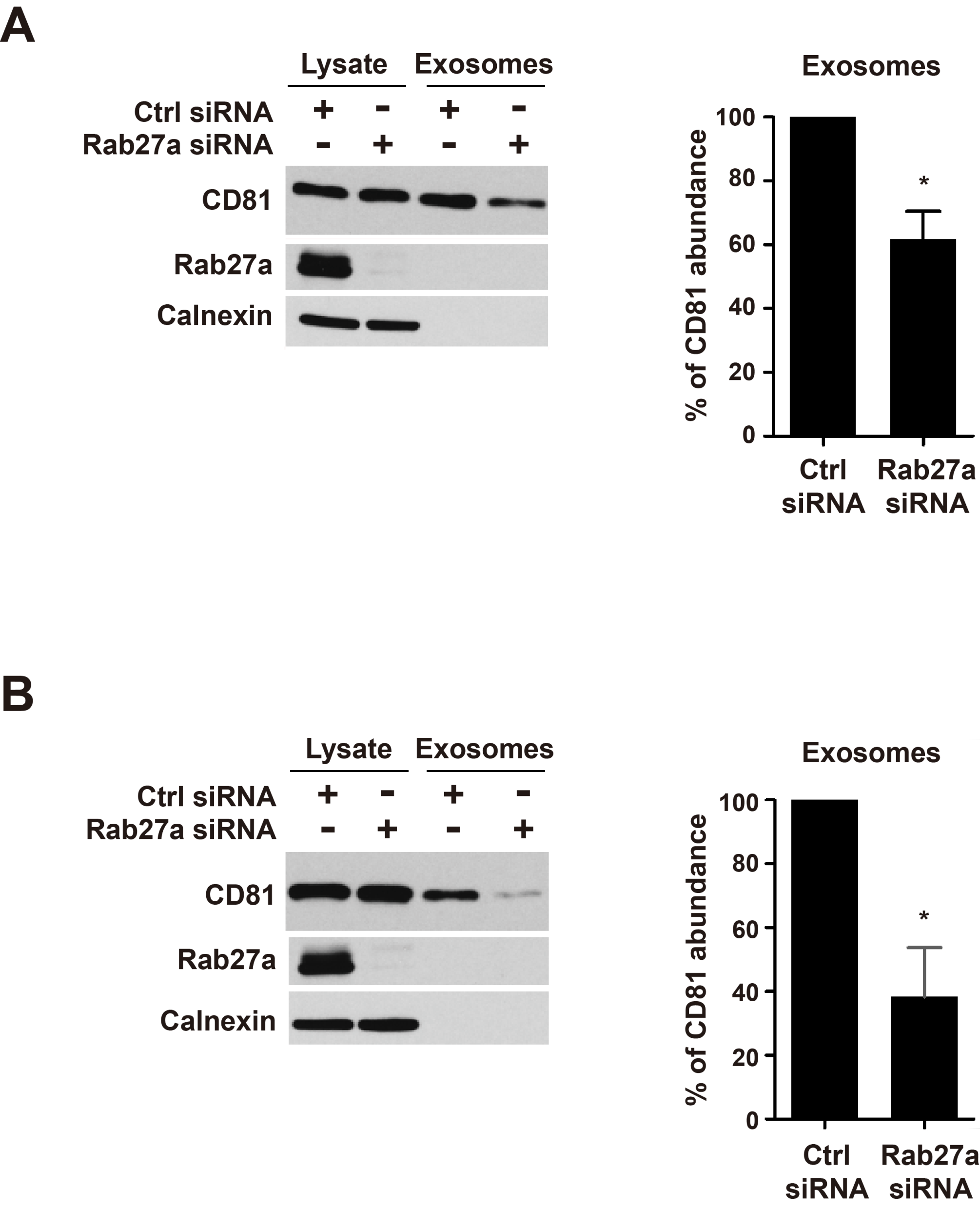

Supplement: S1 Fig — Control and Rab27a siRNA-treated cells were uninfected- (A) or HCV-infected (B). Supernatant was collected at day 3 post-infection from the cell culture medium and subjected to differential centrifugation (see S1 Methods). The resulting pellet (exosome fraction) and cell lysates were analyzed under non-reducing conditions by Western blot for Rab27a, CD81 and Calnexin. Quantification of CD81 protein abundance (right). The data are representative of five independent replicates (*P<0.05, Student’s t-test). (TIF) [file ppat.1005116.s001.tif]

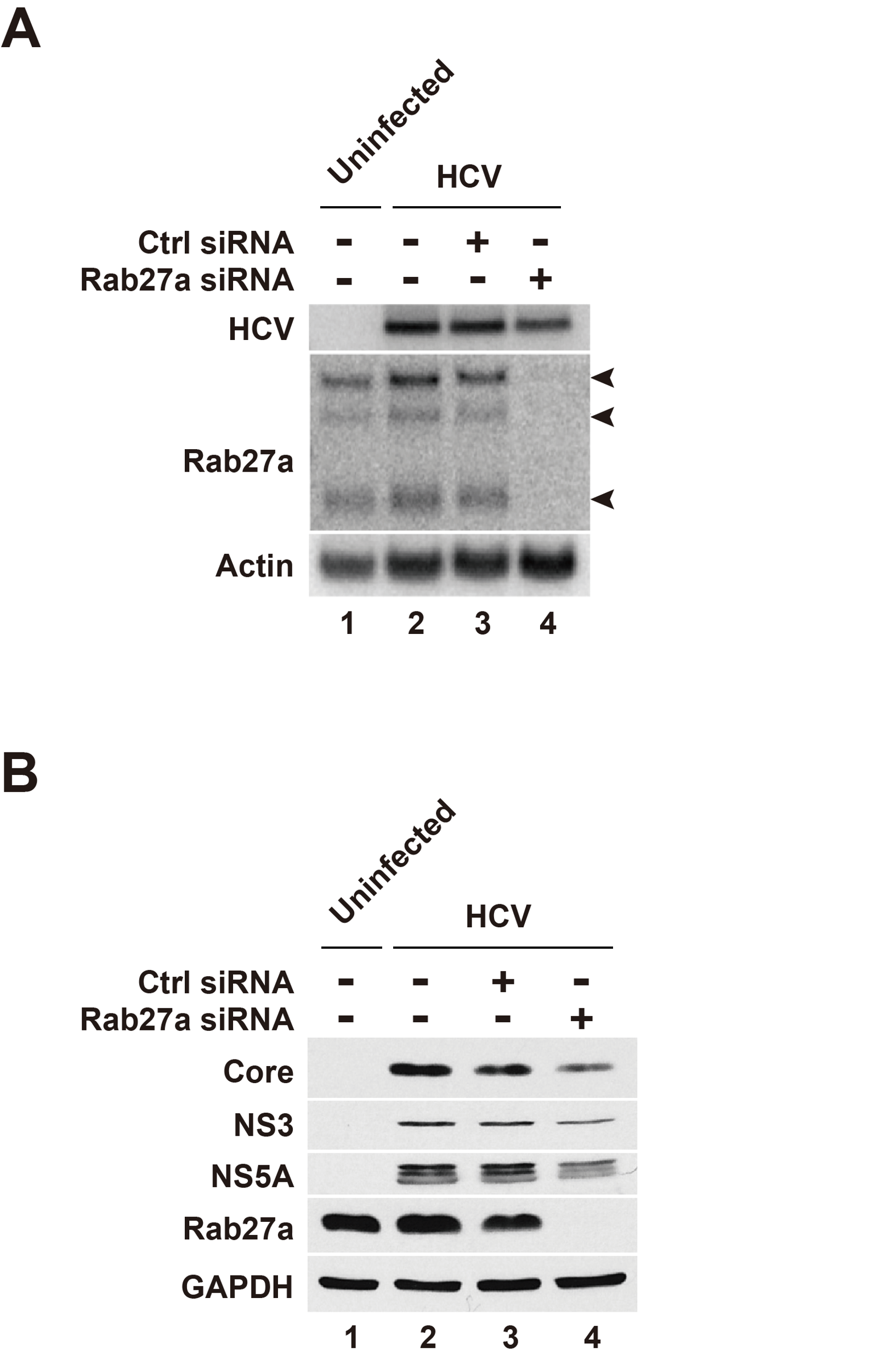

Supplement: S2 Fig — Cells were transfected with siRNAs at day 1 and infected with HCV at MOI = 10 at day 2. Cells were harvested 24 h post-infection. Effects on HCV RNA (A) and protein (B) abundance are shown in Northern and Western blot analyses, respectively. (TIF) [file ppat.1005116.s002.tif]

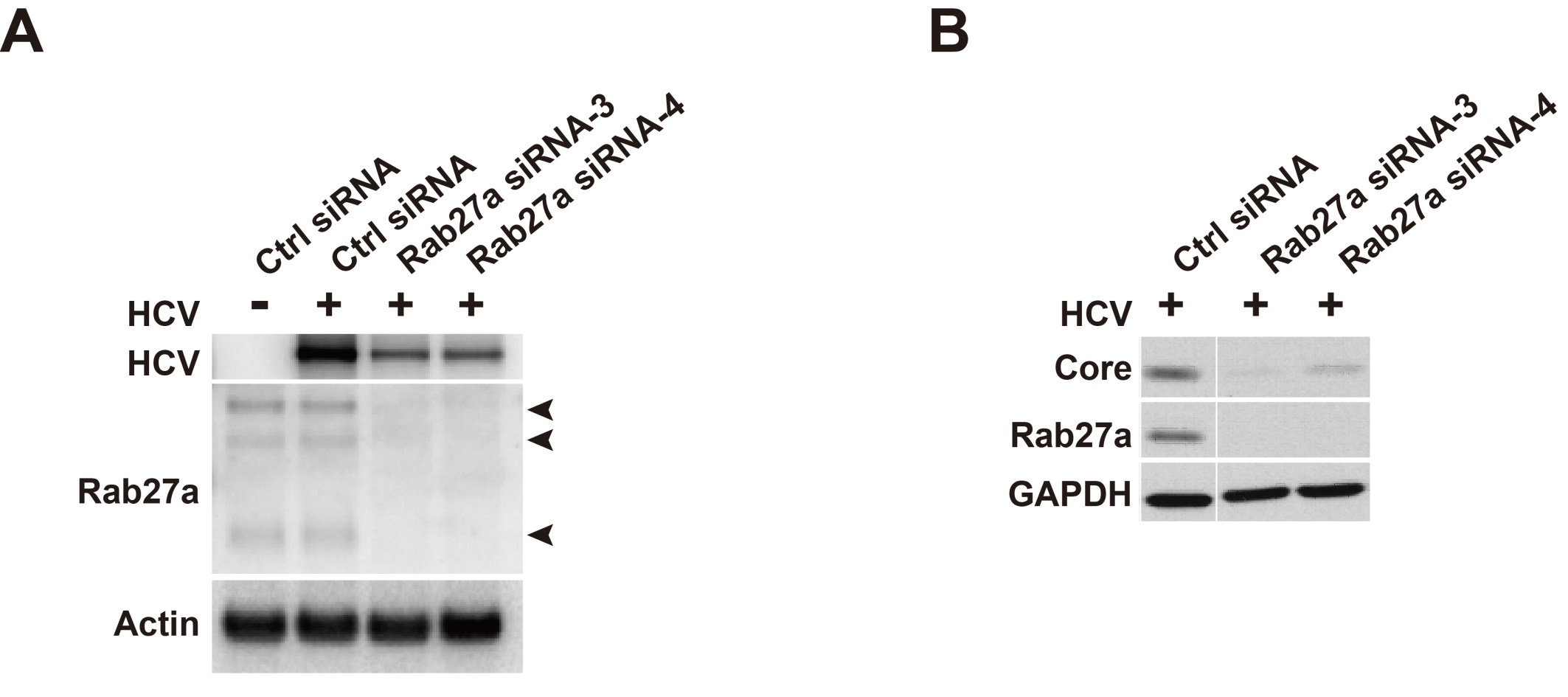

Supplement: S3 Fig — (A) Northern blot analysis of HCV and Rab27a mRNA abundance. Data is representative of at least three independent experiments. (B) Western blot analysis of Rab27a and HCV Core. GAPDH served as a loading control. Immunoblot is representative of three independent experiments. (TIF) [file ppat.1005116.s003.tif]

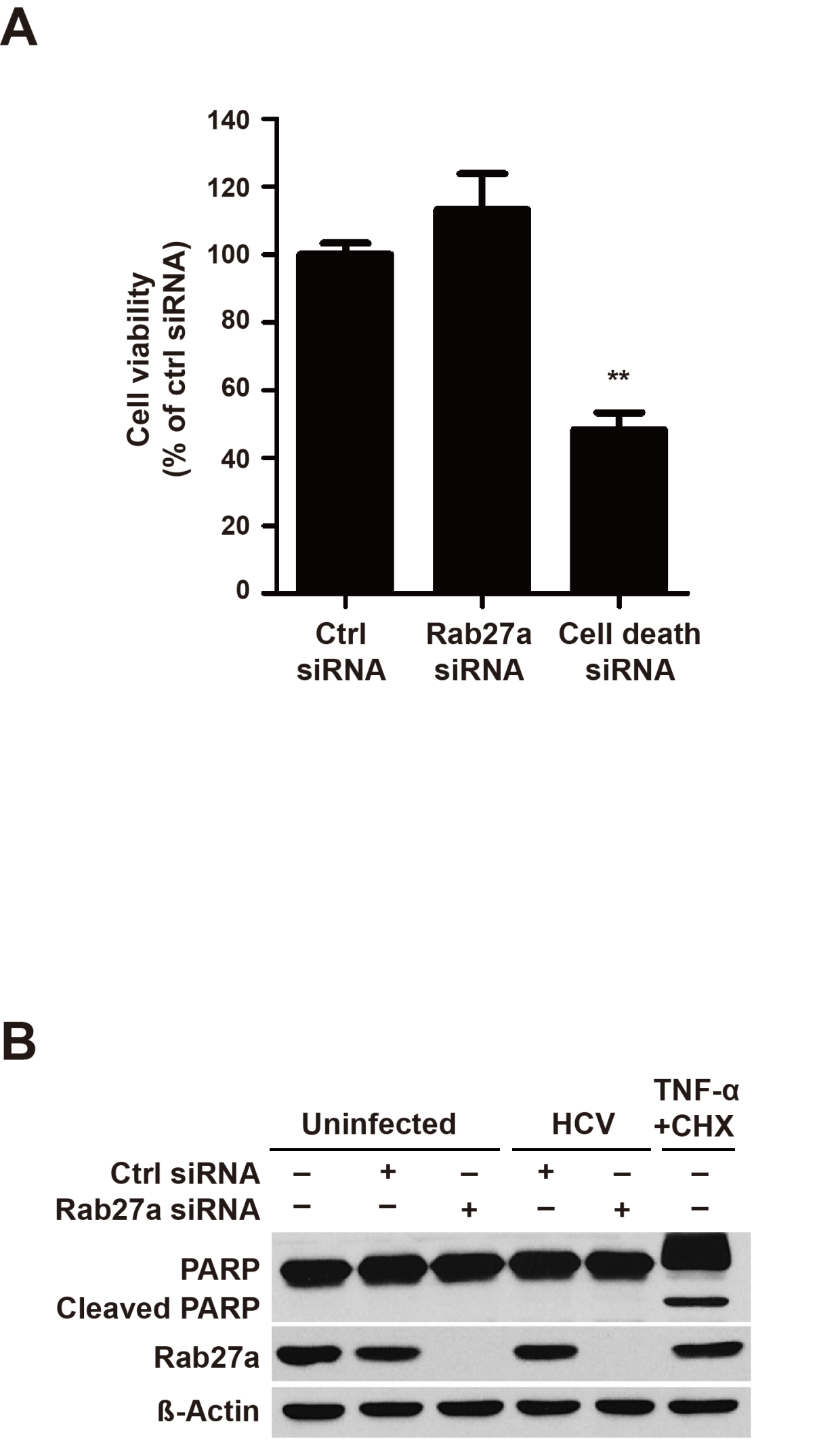

Supplement: S4 Fig — (A) MTT assay of Rab27a siRNA-transfected cells. Control siRNA transfected cells was set to 100%. Cell death siRNA was used as a control for cell viability. The data are representative of four independent experiments (**P<0.005, Student’s t-test). (B) Control or Rab27a siRNA-treated cells were infected with HCV and harvested at day 3 post-infection. Apoptosis induction was assessed by PARP cleavage. Lysate from cells treated with cycloheximide (CHX) at 10 μg/ml and TNF-α at 50 ng/ml for 18 hr was used as a positive control. β-Actin served as loading control. Immunoblot is representative of three independent experiments. (TIF) [file ppat.1005116.s004.tif]

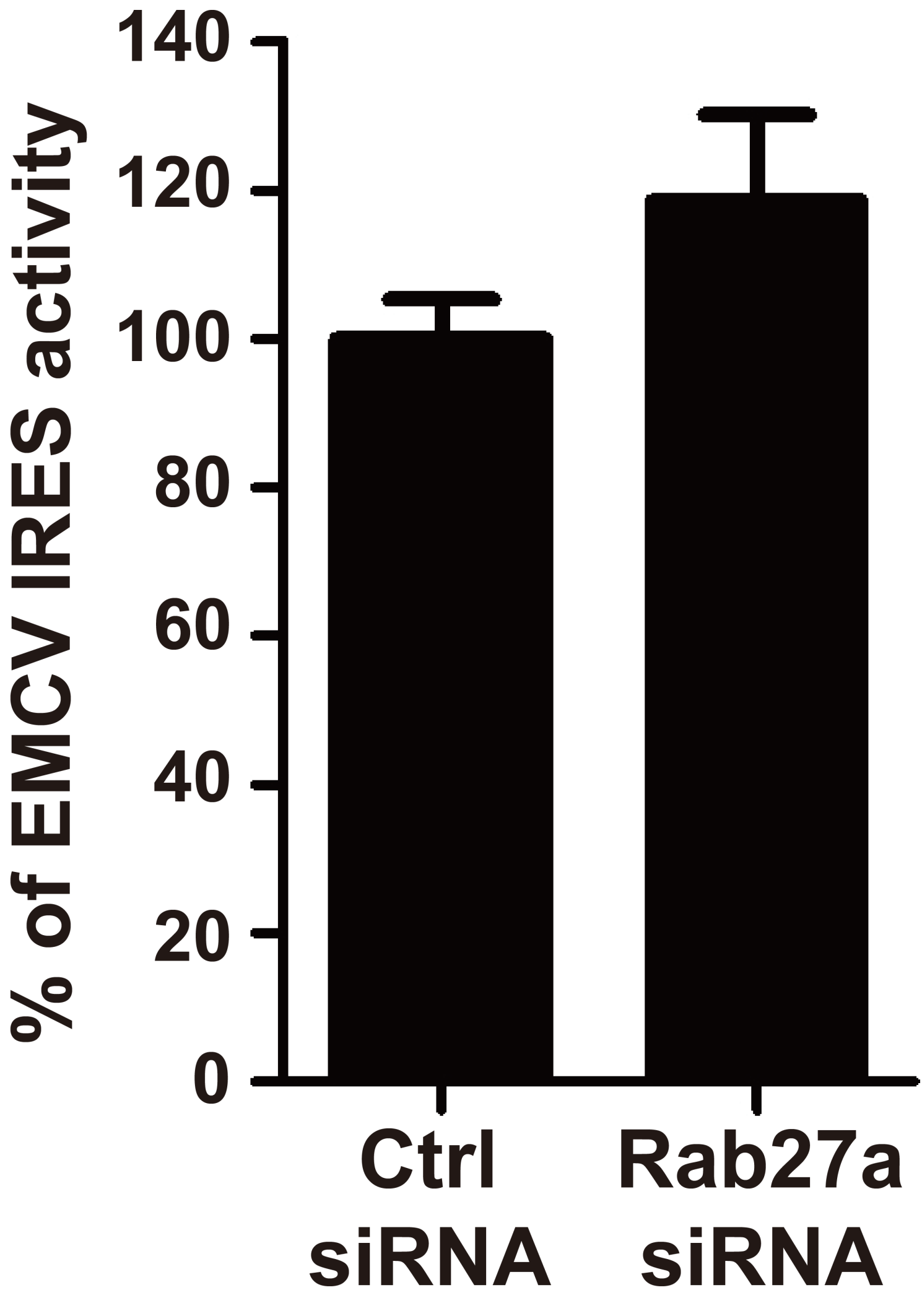

Supplement: S5 Fig — Huh7 cells were transfected with control or Rab27a siRNAs at 50 nM one day prior to pRL-EMCV IRES-FF plasmid transfection. Activities of firefly and Renilla luciferase were measured 24 hours later. The EMCV IRES activity (ratio of firefly luciferase to Renilla luciferase) in control siRNA-transfected cells was set to 100%. The data are representative of three independent experiments. (TIF) [file ppat.1005116.s005.tif]

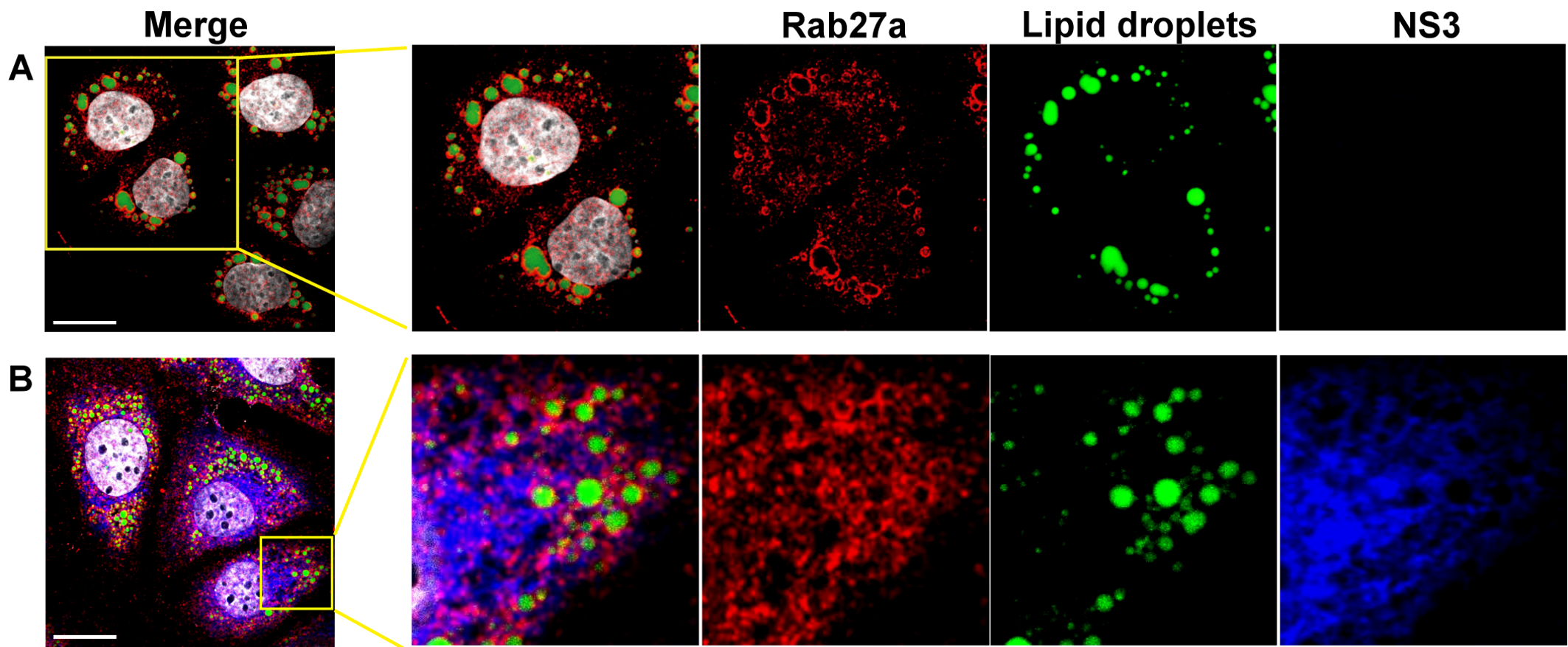

Supplement: S6 Fig — Huh7 cells were uninfected (A) or HCV-infected (B) and then immune-stained for endogenous Rab27a (red) and NS3 (blue). Lipid droplets were stained with Bodipy 493/503 (green) and nuclei were stained with Hoechst 33258 (white). Scale bar, 20 μm. (TIF) [file ppat.1005116.s006.tif]

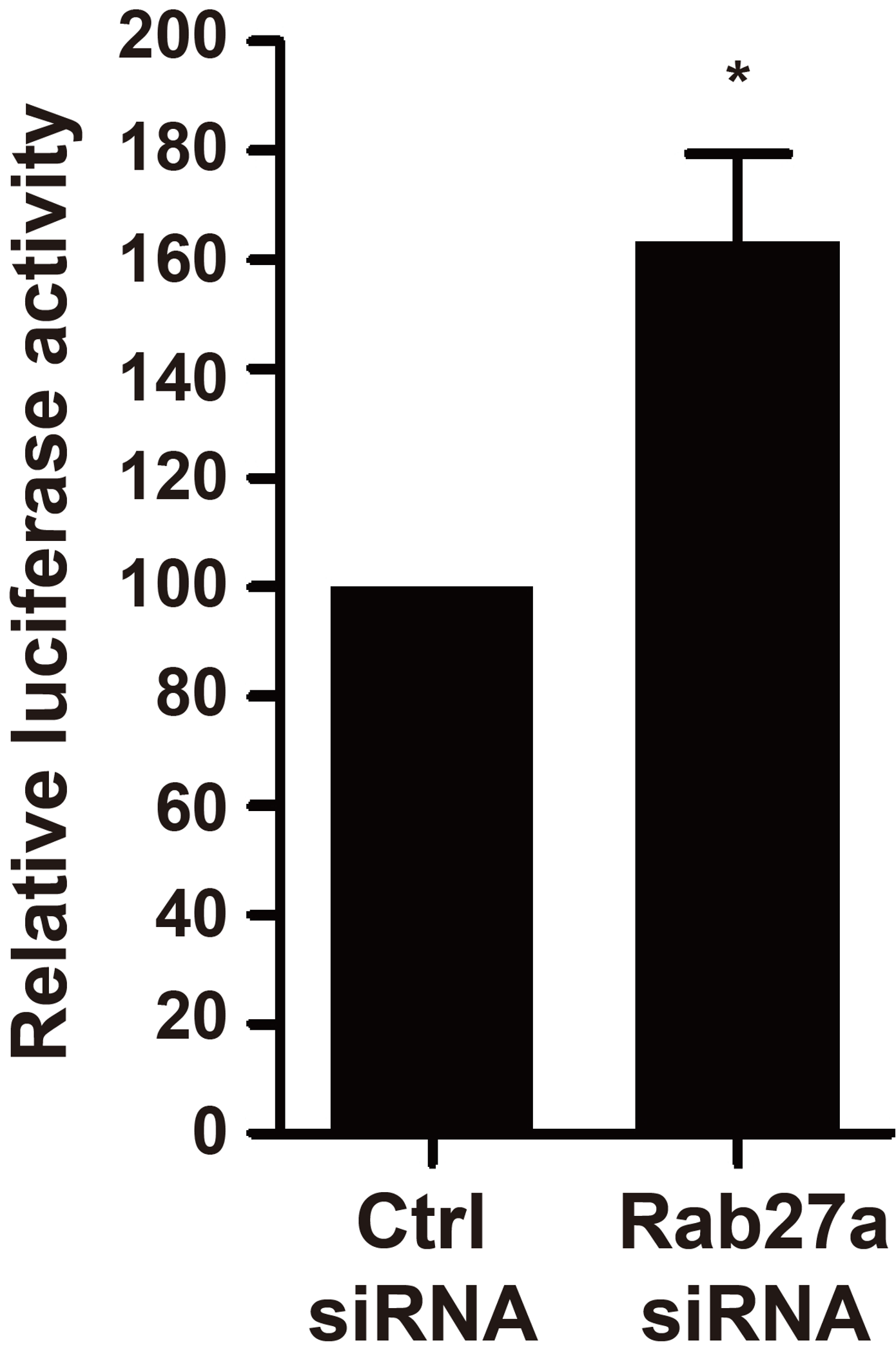

Supplement: S7 Fig — miR-122 activity was determined in control and Rab27a-depleted cells expressing plasmid pLUC-122x2 that transcribes firefly luciferase mRNA which contains miR-122 binding sites in its 3’ noncoding region. The cells were co-transfected with a Renilla reporter plasmid as a transfection control (see S1 Methods). The data are representative of three independent replicates (*P<0.05, Student’s t-test). (TIF) [file ppat.1005116.s007.tif]

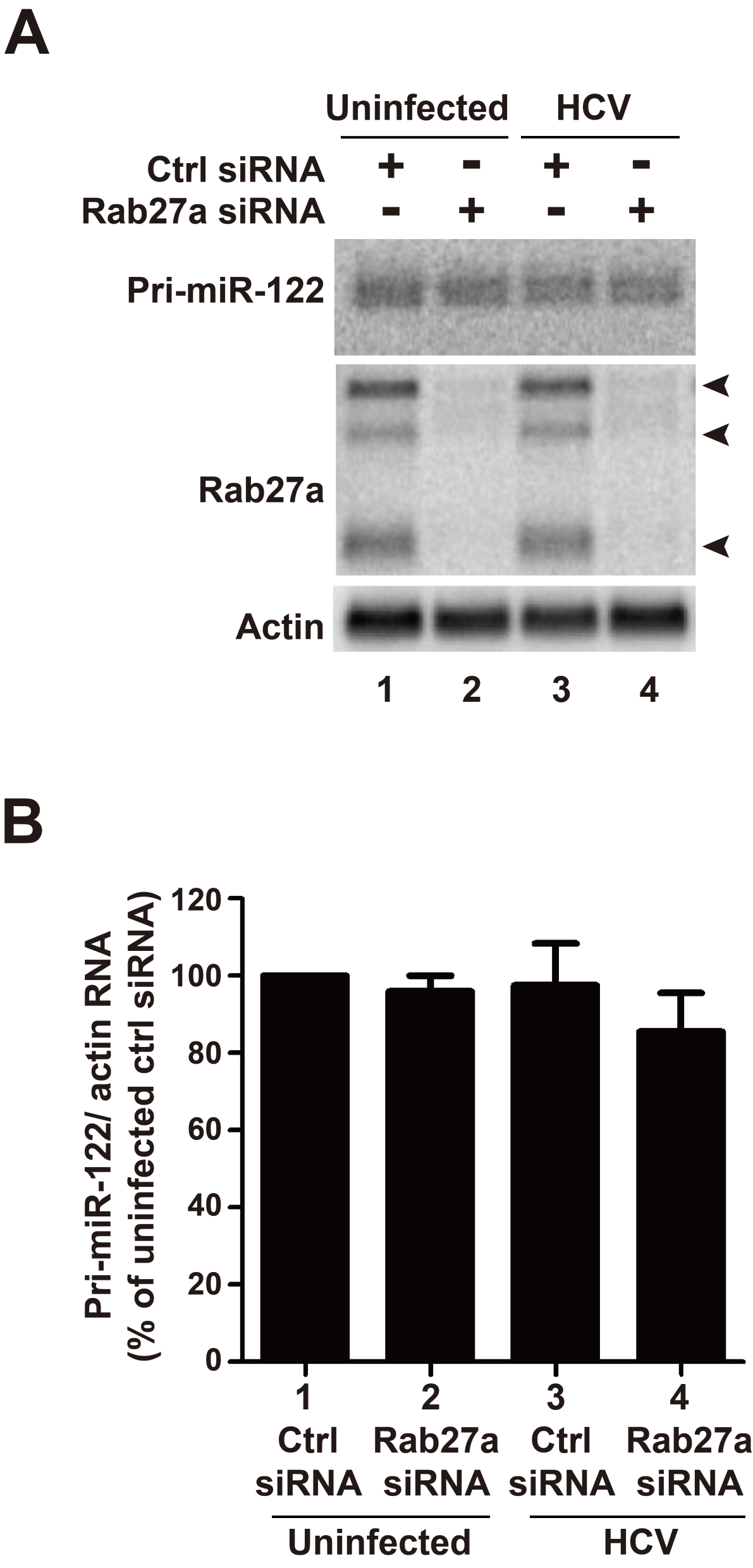

Supplement: S8 Fig — (A) Effect on pri-miR-122 abundance. Control or Rab27a siRNAs-treated cells were uninfected- or HCV-infected. The abundance of pri-miR-122 was measured by Northern blot analysis 3 days post-infection. (B) Quantification of pri-miR-122. Pri-miR-122 was normalized to actin mRNA. Data from control siRNA treated cells was set to 100%. The data are representative of four independent replicates. (TIF) [file ppat.1005116.s008.tif]

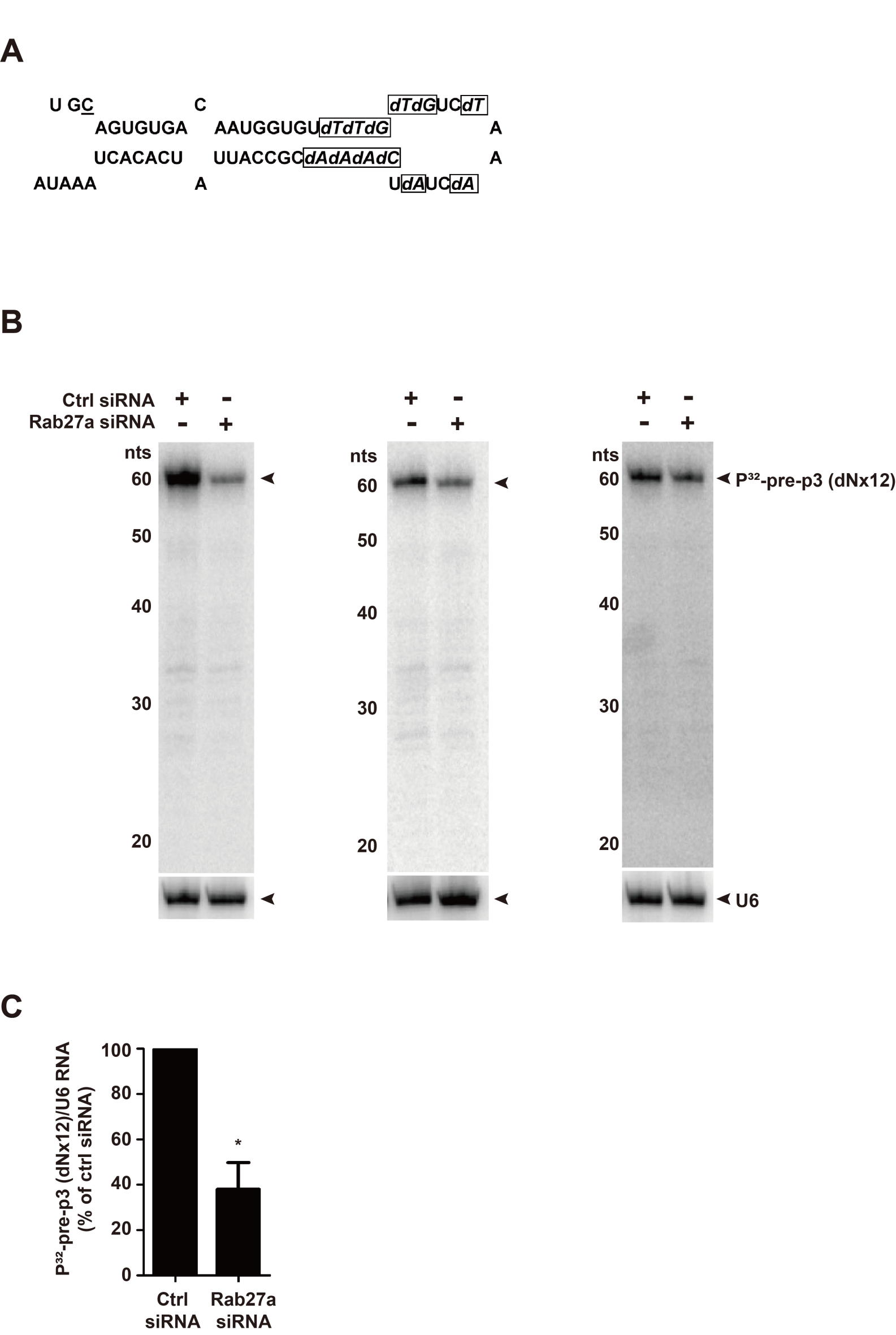

Supplement: S9 Fig — (A) Sequence and predicted structure of Dicer-resistant pre-p3 miR-122(dNx12). The deoxynucleotides are highlighted in a box. The mutated C-nucleotide at position 3 in mature miR-122 is underlined. (B) Effect on pre-p3 miR-122(dNx12). Control and Rab27a siRNA-treated cells were transfected with 5’-32P-labelled pre-p3-miR-122(dNx12). Cells were harvested one day post-transfection. The total RNA containing 5’-32P-labelled pre-p3 miR-122(dNx12) was separated by gel electrophoresis, transferred onto a Hybond-N+ membrane. Autoradiograph of membranes from three independent experiments are shown. To generate a loading control, the membranes were subsequently hybridized with a labelled DNA probe that is complementary to U6 snRNA. Three independent experiments are shown in (B) and quantitated in (C). (TIF) [file ppat.1005116.s009.tif]
