# Supplementary material for: Supporting Role for GTPase Rab27a in Hepatitis C Virus RNA Replication through a Novel miR-122-Mediated Effect
Source: PLoS Pathog. 2015 Aug 25;11(8):e1005116. doi: 10.1371/journal.ppat.1005116 (PMC4549268; doi:10.1371/journal.ppat.1005116)
Supplement: S1 Methods — (DOCX) [file ppat.1005116.s010.docx]

**Supplemental Methods**

**Antibodies and reagents**

The following primary antibodies were used for Western blot analysis: anti-NS3 (Abcam, ab65407), anti-NS5A (a gift from Timothy Tellinghuisen).

**RNA oligonucleotides**

The siRNA sequences are as follow: Rab27a siRNA-3 targeting the 3’ NCR of Rab27a mRNAs, 5’-TGGCACAGTGAGAGATTAAdTdT-3’; Slp4 siRNA-2, 5’-GCGACUAAAGAAUGAGUUAdTdT-3’ (Stanford Pan facility). Rab27a siRNA-4 (SMARTpool, ON-TARGETplus human Rab27a siRNAs) and Slp4 siRNA-1 (SMARTpool, ON-TARGETplus human SYTL4) were purchased from Thermo Scientific (Catalog no. L-004667-00-0005 and L-007111-01-0005). Pre-p3 dNx12 was ordered from Dharmacon (5’-UGCAGUGUGACAAUGGUGUdTdTdGdTdGUCdTAAdACUdAUdCdAdAdACGCCAUUAUCACACUAAAUA).

**Exosome preparation**

Exosomes were prepared according to a previously described method [[12](#_ENREF_12)]. Before the harvest, cell culture medium was replaced with serum-free medium. After incubation for additional 18-20 hours, the supernatant was collected and sedimented for 10 min at 500xg, at 4°C, and for 20 min at 16,500xg, at 4°C. The supernatant was passed through a 0.2 $\mu$m filter, and sedimented for 80 min at 120,000xg, at 4°C. The pellet was resuspended in 2x SDS sample buffer.

**MTT assay**

Huh7 cells (2500 cells) were seeded in a 96-well plate. After overnight incubation, cells were transfected with 50 nM siRNA duplexes using Dharmafect I reagent. The cell viability was determined at day 2 post-transfection using Cell Proliferation Kit I (MTT, Roche) following the manufacturer’s instruction. As a positive control, cell viability was measured after treatment with 50 nM AllStars Hs Cell Death Control siRNA (Qiagen). The percentage of cell viability was calculated as the ratio of absorbance in Rab27a siRNA-treated cells compared with that in control treated cells. All experiments were performed in triplicate and repeated in four independent experiments.

**PARP Western blot**

Cells were transfected with siRNAs and infected with HCV at one day post-transfection. The infected cells were harvested at day 3 post-infection, and Western Blot was performed as described in Experimental Procedures. An anti-PARP primary antibody (Cell Signaling 9542) was employed in Western blot analysis.

**Luciferase-based microRNA assay**

Plasmid pLUC-122x2 and pSV40-RL have been previously described [[3](#_ENREF_3)]. Plasmid pLUC-122x2, contains two copies of HCV sequence 1-45 (with miR-122 binding sits) in the 3’ NCR of the firefly luciferase gene. Control and Rab27a-depleted cells were transfected in 6 mm dishes with firefly luciferase (pLUC-122x2) and Renilla luciferase (pSV40-RL) plasmids at 1 μg and 0.1 μg, respectively, using Lipofectamine 2000 following the manufacturer’s instruction. Cells were lysed in 1x Passive buffer (Promega) at 24 h post-transfection. The luciferase activities were determined using the Dual-Luciferase Reporter Assay System (Promega), following the manufacturer’s instruction. The Renilla luciferase plasmid was used as a control for transfection efficiency, and the firefly activity from the sample was normalized to Renilla luciferase expression.

**Detection of Pri-miR-122 RNA**

Total RNA was isolated from Huh7 cells using Trizol following the manufacturer’s recommendations. Pri-miR-122 RNA was enriched after purification of total RNA on OligoTex-beads (Qiagen). RNA was resolved by electrophoresis in 1 % agarose gels. The abundance of pri-miR-122 was measured by Northern blot analysis as described in the previous Method section. The α-^32^P dATP-RadPrime DNA labeled probe (Invitrogen) complementary to the sequence of human precursor miR-122 was used to detect pri-miR-122 transcript.

**Detection of pre-p3 (dNx9)**

The 5’ end of pre-p3 (dNx9) RNA was labeled with ^32^P using T4 polynucleotide kinase (PNK; NEB) for 1 h at 37℃ in a total volume of 20 $\mu$L containing 10 pmol RNA, 1$\times$ PNK buffer, 150 $\mu Ci \gamma$-^32^P ATP, 20 units SUPERase In RNase inhibitor (Ambion) , and 10 units PNK. The ^32^P-labelled RNA was polyacrylamide gel electrophoresis purified, and eluted in 20 $\mu$L RNase-free water. Cells were transfected with 50 nM of siRNA duplexes using Dharmafect I reagent, transfected again with siRNA duplexes at day 3, transfected with 2 $\mu$L purified 5’ end ^32^P-labelled pre-p3 RNA (~0.4 pmol, about 12000 cpm) at day 4, and harvested at 24 h post-transfection (day 5). Total RNA was extracted using TRIzol following the manufacturer’s protocol, separated by electrophoresis in 12% denaturing urea polyacrylamide gels, and transferred onto Hybond-N+ membranes. To detect 5’-^32^P-labelled pre-p3 (dNx12), the blot was exposed to a phosphor screen. U6 snRNA was detected using Northern blot analysis described in Materials and Methods.
